# ZBTB2 represses HIV-1 transcription and is regulated by HIV-1 Vpr and cellular DNA damage responses

**DOI:** 10.1371/journal.ppat.1009364

**Published:** 2021-02-26

**Authors:** James W. Bruce, Megan Bracken, Edward Evans, Nathan Sherer, Paul Ahlquist

**Affiliations:** 1 Rowe Center for Research in Virology, Morgridge Institute for Research, Madison, Wisconsin, United States of America; 2 Institute for Molecular Virology, University of Wisconsin, Madison, Wisconsin, United States of America; 3 McArdle Laboratory for Cancer Research, University of Wisconsin, Madison, Wisconsin, United States of America; 4 Howard Hughes Medical Institute, University of Wisconsin, Madison, Wisconsin, United States of America; 5 Laboratory for Optical and Computational Instrumentation, University of Wisconsin, Madison, Wisconsin, United States of America; Institut Cochin, INSERM U1016, FRANCE

## Abstract

Previously, we reported that cellular transcription factor ZASC1 facilitates DNA-dependent/RNA-independent recruitment of HIV-1 TAT and the cellular elongation factor P-TEFb to the HIV-1 promoter and is a critical factor in regulating HIV-1 transcriptional elongation (PLoS Path e1003712). Here we report that cellular transcription factor ZBTB2 is a novel repressor of HIV-1 gene expression. ZBTB2 strongly co-immunoprecipitated with ZASC1 and was dramatically relocalized by ZASC1 from the cytoplasm to the nucleus. Mutations abolishing ZASC1/ZBTB2 interaction prevented ZBTB2 nuclear relocalization. We show that ZBTB2-induced repression depends on interaction of cellular histone deacetylases (HDACs) with the ZBTB2 POZ domain. Further, ZASC1 interaction specifically recruited ZBTB2 to the HIV-1 promoter, resulting in histone deacetylation and transcription repression. Depleting ZBTB2 by siRNA knockdown or CRISPR/CAS9 knockout in T cell lines enhanced transcription from HIV-1 vectors lacking Vpr, but not from these vectors expressing Vpr. Since HIV-1 Vpr activates the viral LTR by inducing the ATR kinase/DNA damage response pathway, we investigated ZBTB2 response to Vpr and DNA damaging agents. Expressing Vpr or stimulating the ATR pathway with DNA damaging agents impaired ZASC1’s ability to localize ZBTB2 to the nucleus. Moreover, the effects of DNA damaging agents and Vpr on ZBTB2 localization could be blocked by ATR kinase inhibitors. Critically, Vpr and DNA damaging agents decreased ZBTB2 binding to the HIV-1 promoter and increased promoter histone acetylation. Thus, ZBTB2 is recruited to the HIV-1 promoter by ZASC1 and represses transcription, but ATR pathway activation leads to ZBTB2 removal from the promoter, cytoplasmic sequestration and activation of viral transcription. Together, our data show that ZASC1/ZBTB2 integrate the functions of TAT and Vpr to maximize HIV-1 gene expression.

## Author summary

The Human immunodeficiency virus 1 (HIV-1) TAT and VPR proteins, in combination with cellular transcription factors, regulate the switch between transcriptionally active productive infection and the transcriptionally inactive latent state. Previously we reported that ZASC1, a cellular transcription factor linked to multiple squamous cell carcinomas and inherited ataxias, contributes to an RNA-independent, DNA-dependent step in recruiting the TAT/P-TEFb complex that is critical for HIV-1 transcription elongation to the HIV-1 promoter. Here we show ZASC1 interacts with ZBTB2, another cellular transcription factor with strong links to cancer. ZASC1 interaction relocalizes ZBTB2 from the cytoplasm to the HIV-1 promoter in the nucleus where ZBTB2 interacts with cellular HDACs, increases HIV-1 promoter histone deacetylation and represses viral transcription. We show that Vpr-mediated activation of the ATR/DNA damage pathway regulates ZBTB2 relocalization by ZASC1. Thus, the cellular transcription factors ZASC1 and ZBTB2 regulate the transcription elongation activities of HIV-1 TAT and the Vpr activation of the cellular DNA damage response pathway to determine the transcriptional fate of the HIV-1 provirus. These results also have strong implications for the role of ZASC1/ZBTB2 and the DNA damage response in cancer and inherited ataxias.

## Introduction

Human immunodeficiency viruses type-1 and 2 (HIV-1 and HIV-2) are the causative agents of acquired immune deficiency syndrome (AIDS). After HIV entry, the viral RNA is reverse transcribed into double stranded DNA, traffics to the nucleus and is integrated into the cellular chromosome. The resulting provirus is transcribed by host RNA polymerase II (pol II) from the unique 3’ (U3) element in the viral long terminal repeat (LTR).

The LTR promoter contains many overlapping binding sites for cellular transcription factors that differentially modulate expression depending on cell type and in response to multiple signaling pathways [1,2]. In addition to cellular factors, HIV-1 transcription is regulated by the viral TAT and Vpr proteins. The HIV-1 promoter efficiently initiates transcription but pol II stalls after extending approximately 100 nucleotides [3]. This block in elongation is overcome by the viral TAT protein and a structured RNA element in the nascent mRNA known as the transactivation response region (TAR). TAT recruits the cellular transcriptional elongation factor P-TEFb to TAR, resulting in phosphorylation of the negative elongation factor (NELF), DRB sensitivity inducing factor (DSIF) and the C-terminal domain (CTD) of pol II by P-TEFb, releasing the stalled polymerase and dramatically increasing transcription elongation [4,5].

Viral protein R (Vpr) increases HIV-1 gene expression [6] synergistically with HIV-1 TAT [7, 8]. Vpr is a multifunctional virion protein that is delivered into the cell upon viral entry and has been reported to contribute to uncoating, reverse transcription, and nuclear import [9,10]. One of the most studied functions of virion-delivered Vpr is its induction of G2 arrest. Vpr induces G2 arrest by stimulating the DNA damage response (DDR) through activating the ATR kinase pathway. Most current models for this G2 arrest rely on Vpr commandeering the proteasome to degrade host proteins involved in chromosome maintenance, including the structure specific endonuclease (SSE) regulator SLX4 complex, histone deacetylases and minichromosome maintenance 10 DNA replication factor [11–18]. The biological significance of this arrest is not well understood, but it is clear that the HIV-1 LTR promoter is significantly more active in G2 phase, resulting in more virus production [6–8]. Indeed, either chemical inhibition of ATR kinase or siRNA knockdown of ATR decreased stimulation of transcription by Vpr [19,20]. Conversely, activation of ATR by DNA damage increases HIV-1 transcription [21–24]. Further, Vpr activation of the DDR may facilitate HIV-1 avoidance of innate immune sensing [25,26].

Cellular transcription factors, TAT, and Vpr regulate the switch between productive infection and the transcriptionally inactive state of the provirus known as latency [27,28]. Current highly active anti-retroviral therapy (HAART) targets viral enzymes and is ineffective at clearing this reservoir of latently infected cells. Ongoing reactivation of virus from latently infected cells necessitates long term adherence to HAART [29]. Insufficient TAT expression can lead to viral silencing and establishment of latency, while, conversely, TAT expression forms a positive feed-back loop that is essential for reactivation [30,31]. Similarly, exogenous Vpr expression or simulating Vpr expression by activating the ATR pathway by DNA damaging agents efficiently reactivates latent proviruses [21,32–36]. Better understanding of the interactions between TAT, Vpr and cellular transcription factors during productive and latent infection could lead to therapies that either inhibit reactivation or specifically stimulate reactivation followed by viral clearance by HAART [37].

Previously we reported that the cellular transcription factor zinc finger associated with squamous cell carcinomas (ZASC1) is a novel regulator of HIV-1 transcription [38]. Copy number amplification of the chromosomal region containing ZASC1 is linked to multiple squamous cell carcinomas, an increased propensity for metastasis [39–42], and has been associated with inherited ataxias [43]. Furthermore, ZASC1 has been reported to contribute to β-catenin nuclear transport [44] and is a sequence-specific DNA binding protein that binds to and activates the murine leukemia virus (MLV) U3 promoter [45].

We showed that ZASC1 binds to highly conserved DNA elements in the HIV-1 LTR just upstream of the TAR element and regulates proviral transcription by stimulating HIV-1 TAT activity [38]. Furthermore, we found that ZASC1 recruits TAT and P-TEFb to the HIV-1 promoter in the presence and absence of TAR. Thus, ZASC1 contributes to an RNA-independent, DNA-dependent step in recruiting to the HIV-1 promoter the TAT/P-TEFb complex that is a critical factor in promoting HIV-1 transcription elongation [38].

Recently, several immunoprecipitation—mass spectroscopy experiments on diverse sets of large transcription complexes, including hormone receptor co-activator complexes [46], SET1/MLL histone methyltransferase complexes [47] and HDAC corepressor complexes [48], co-purified ZASC1 and another cellular transcription factor, ZBTB2. ZBTB2 is a POK (POZ and Krüppel) transcription factor with an N-terminal POZ domain and 4 C-terminal Krüppel-like C_2_H_2_ zinc fingers. POZ domains are a conserved protein interaction motif that often bind transcriptional co-repressors [49,50]. ZBTB2 has been reported to repress several cellular promoters including key regulators of the p53 DNA damage pathway [51,52]. Similar to ZASC1, ZBTB2 has strong links to cancer [53–55]. In this study, we explored the ZASC1 and ZBTB2 interaction and the functional implications of this interaction for HIV-1 gene expression. We show that ZASC1 binds ZBTB2 and recruits ZBTB2 to the HIV-1 promoter. Further, once ZBTB2 is bound to the HIV-1 promoter it recruits cellular HDACs and represses HIV transcription. Importantly, we show that the interaction between ZASC1 and ZBTB2 is regulated by Vpr-mediated activation of the ATR/DNA damage pathway. Taken together, our data show that ZASC1/ZBTB2, HIV-1 TAT and Vpr form a regulatory nexus with the cellular DNA damage response pathway.

## Results

### ZASC1 and ZBTB2 interact

**Fig 1:**
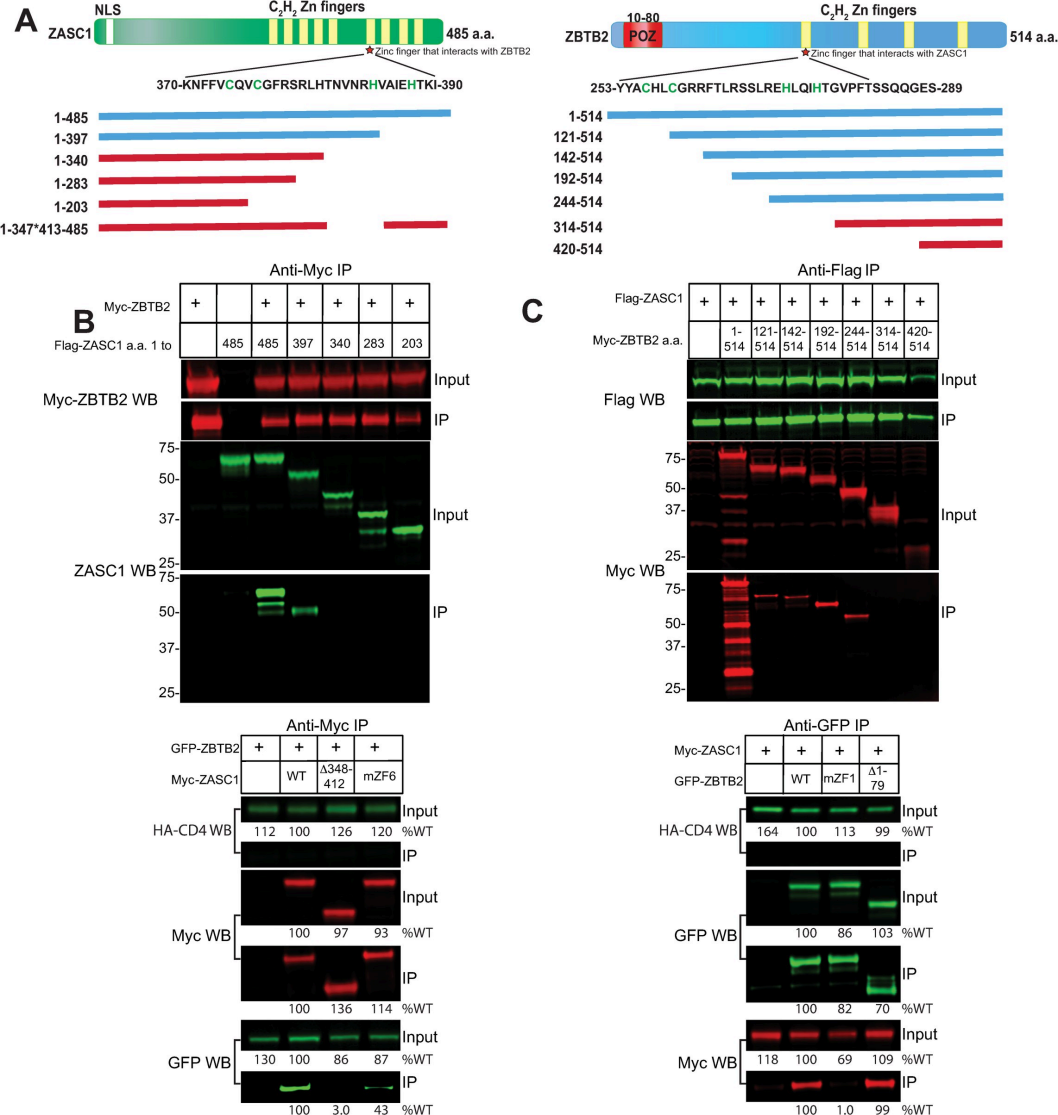
ZASC1 and ZBTB2 interact. (A) Schematic of ZASC1 and ZBTB2 and relative location of N-terminal and internal deletion variants tested for interaction. Blue indicates interaction and red indicates a failure to interact. The location of zinc fingers in each protein are indicated in yellow. Sequences of the interaction domains in ZASC1 zinc finger 6 (ZF6) and ZBTB2 zinc finger 1 are shown below the respective protein maps, with Cys and His residues implicated in Zn^2+^ coordination indicated in green. The ZASC1 mZF6 variant has C376, C379S,H392A, H397A mutations while the ZBTB2 mZF1 variant has C256S, C259S, H272A, H276A mutations. HEK293 cells (1X10^7^) were transfected with expression plasmids encoding the epitope tagged forms of the indicated proteins. 48 h post-transfection, cells were lysed, and epitope tagged proteins were immunoprecipitated (IP). Starting material and IP material was separated by SDS-PAGE and analyzed by western blotting (WB) using the indicated antibodies as described in materials and methods. (B) Co-immunoprecipitation of ZASC1 variants by WT ZBTB2. (C) Co-immunoprecipitation of ZBTB2 variants by WT ZASC1. Numbers to the left of graphs indicate position of molecular weight markers in kDa. Numbers below the graph indicate relative band intensity relative to the well expressing WT ZASC1 and WT ZBTB2. In B and C, no appreciable signal from the HA-CD4 control protein IP was detected (less than 0.1% of input).

To confirm that ZASC1 and ZBTB2 interact [46–48], and to determine the regions of the proteins necessary for this interaction, we undertook a large-scale mapping experiment using co-transfected plasmids encoding Myc-tagged ZBTB2 with plasmids encoding Flag-tagged ZASC1. Micrococcal nuclease was included in all immunoprecipitations to eliminate any contributions of DNA or RNA to the interactions. The findings of these co-immunoprecipitation mapping experiments are summarized in Fig 1A. Full length ZASC1 co-immunoprecipitated with ZBTB2 (Fig 1B), but binding to ZBTB2 was lost with C-terminal truncations after a.a. 340 (Fig 1B upper panel). Subsequent experiments showed that a deletion variant of ZASC1 that lacks ZASC1 a.a. 348 to 412 did not interact with ZBTB2 (Fig 1B lower panel). Since this region encompasses ZASC1 Zinc finger 6 (ZF6) we tested a variant of ZASC1 with the critical C2H2 residues in ZF6 (C376,C379S,H392A, H397A) mutated as shown in Fig 1A. The Zinc Finger 6 (mZF6) variant showed consistently reduced interaction with ZBTB2 (43 ± 2% of WT; Fig 1B lower panel). This data suggests that the 64 a.a. between 348 and 412 contain the ZBTB2 interaction site and that mZF6 contributes to, but is not solely responsible for ZBTB2 interaction with ZASC1.

Similarly, full length ZBTB2 co-immunoprecipitated with ZASC1 (Fig 1C) but this binding was lost with N-terminal truncations beyond a.a. 244 (Fig 1C upper panel). Importantly, while ZBTB2 interaction with ZASC1 is robust, we see no pulldown of ZBTB2 under the same immunoprecipitation conditions in the absence of ZASC1 (see S3A lanes 6 and 7 and S3B lanes 1 and 2 Fig). Subsequent analysis showed that mutations that delete ZBTB2 Zinc finger 1 (ZF1) or specifically mutate the C2H2 residues in ZF1 (C256S, C259S, H272A, H276A) as shown in Fig 1A no longer interacted with ZASC1 (Figs 1C lower panel and S1). Further, a mutant deleted for the transcriptionally relevant POZ domain (deletion of a.a. 1–79) retained interaction with ZASC1. As expected, no immunoprecipitation was observed with transfected control protein HA tagged CD4 (Fig 1B and 1C, lower panels; less than 0.1% of input recovered, vs between 10 and 30% of input recovered for ZBTB2 and ZASC1 immunoprecipitations). Together, these data show that a 64 a.a. region around ZF6 of ZASC1 and ZF1 of ZBTB2 are required to mediate an interaction between the proteins.

### ZASC1 interaction regulates ZBTB2 localization

**Fig 2:**
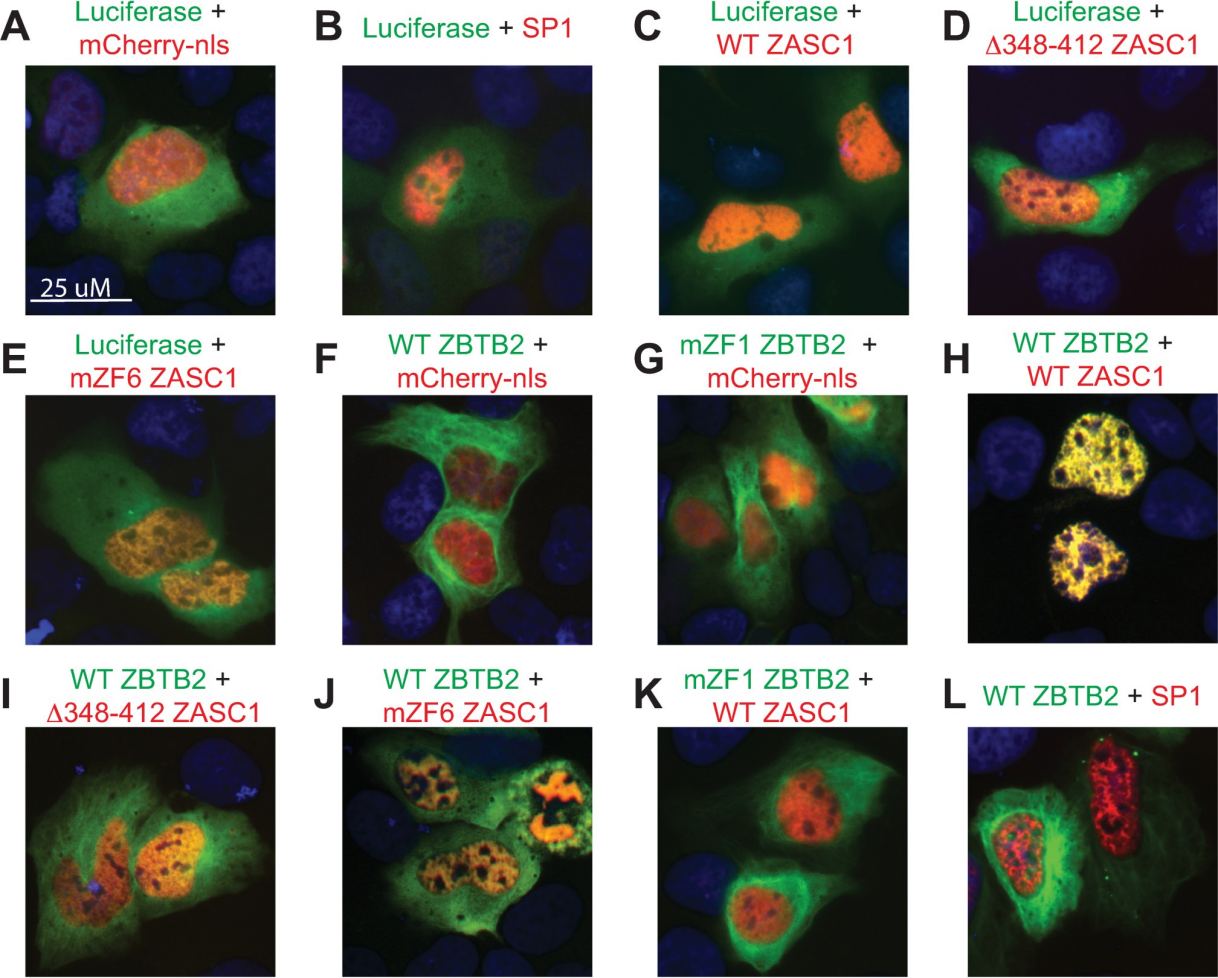
ZASC1 regulates ZBTB2 nuclear localization. Fluorescent images of U2OS cells transfected with expression vectors encoding the indicated mCherry-fusion proteins (mCherry with a nuclear localization signal, SP1 and ZASC1 variants) and GFP- fusion proteins (firefly luciferase and ZBTB2 variants). Nuclei were stained with DAPI. Scale bar applies to all panels.

While exploring the interactions between ZASC1 and ZBTB2 we wanted to determine if both proteins were in the nucleus. We examined this localization in U2OS cells because they have been extensively used in HIV imaging studies [18,56,57]. Similar results were obtained with other common adherent cell lines such as HeLa and HEK293T. U2OS cells were transfected with mCherry and GFP-fusion proteins. In control experiments, GFP-luciferase and mCherry fused to a nuclear localization signal (NLS) were used to mark the cytoplasm and nucleus, respectively (Fig 2A). As a further control, the cellular transcription factor SP1 was fused to mCherry and localized exclusively to the nucleus (Fig 2B). ZASC1 has a strong nuclear localization signal at its N-terminus and has previously been reported to be constitutively nuclear [40,44,45]. Consistent with this, a ZASC1-mCherry fusion was exclusively nuclear (Fig 2C). Deleting the ZBTB2 interaction site ZASC1 a.a. 348 to 412 or mutating ZASC1’s ZF6 had no effect on nuclear localization (Fig 2D and 2E). ZBTB2 has no predicted nuclear localization signal and, in sharp contrast to ZASC1, GFP fusion proteins of either WT ZBTB2 or a ZBTB2 variant with the ZASC1 interaction determinant ZF1 mutated primarily localized to the cytoplasm (Fig 2F and 2G). Strikingly, when both mCherry-ZASC1 and GFP-ZBTB2 were co-expressed, ZBTB2 was dramatically relocalized from the cytoplasm to the nucleus (Fig 2H). This relocalization was dependent on having a WT ZBTB2 interaction domain (a.a. 348–412) in ZASC1 (Fig 2I). Interestingly, mutation of ZF6 in ZASC1 was also sufficient to markedly inhibit ZBTB2 nuclear localization (Fig 2J), suggesting that even though the reduction in immunoprecipitation efficiency was only ~2-fold (Fig 1B lower panel), ZASC1 ZF6 may contribute significantly to stable interaction with ZBTB2 in vivo. Similarly, ZBTB2 required a WT ZASC1 interaction domain in ZBTB2 ZF1 (Fig 2K) for ZASC1-mediated nuclear localization. Importantly, expression of cellular transcription factor SP1, which has been reported to interact with ZBTB2 [51], did not cause detectable nuclear relocalization of ZBTB2 (Fig 2L). Thus, ZBTB2 specifically depends on interaction with ZASC1 for nuclear localization.

### ZBTB2 represses HIV-1 gene expression in the absence of Vpr

**Fig 3:**
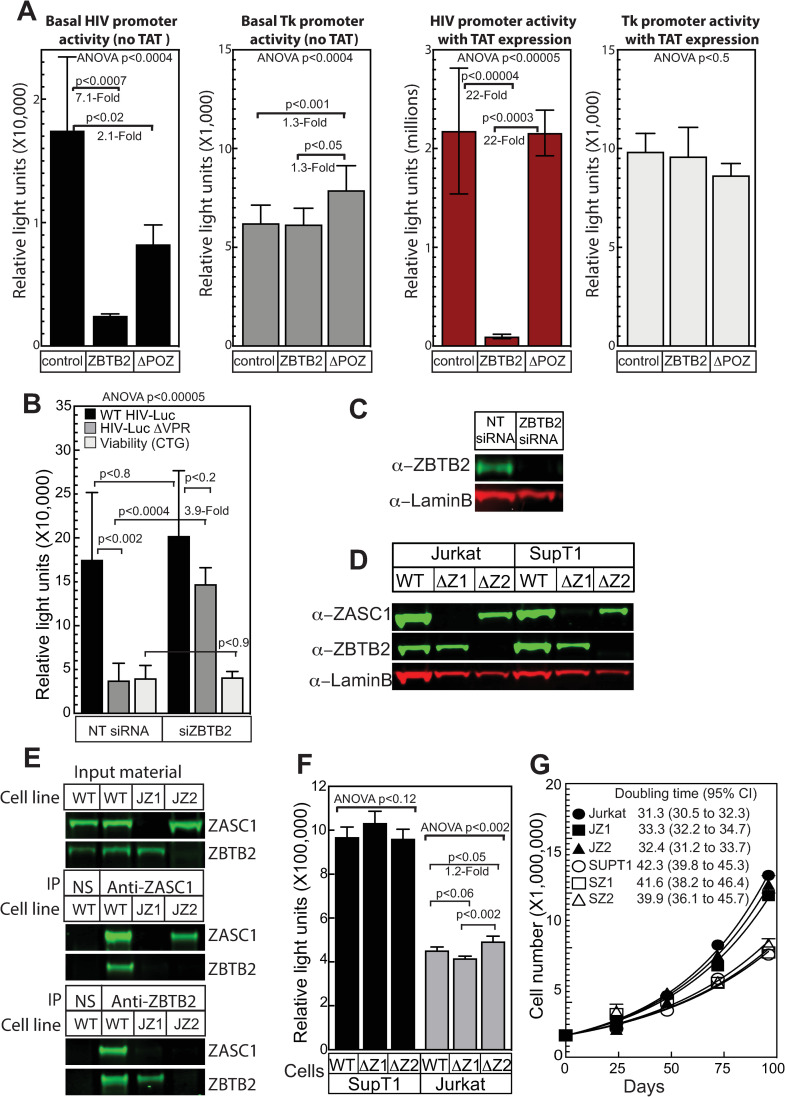
ZASC1 and ZBTB2 regulate HIV-1 gene expression. (A) Jurkat cells were transfected with a HIV-1 promoter-driven reporter plasmid upstream of gaussian luciferase and the herpes simplex virus tk promoter upstream of cypridinia luciferase and the protein encoding plasmids indicated at the bottom. The effects on the basal and TAT activated expression of the WT HIV-1 LTR promoter and the control tk promoter in the presence or absence of ZBTB2 or a ZBTB2 variant with a deletion in the POZ domain (a.a. 1–79, ΔPOZ) are shown). The data shown are the average mean chemiluminescent reporter values obtained in an experiment performed with quadruplicate samples and are representative of three independent experiments. (B) SUPT1 cells transfected with siRNAs targeting ZBTB2 or non-targeting siRNA. Two days post transfection, cells were challenged with VSV-G pseudotyped HIV-1 vectors with non-sense mutations in envelope, and Vpr with firefly luciferase in the NEF locus. Infections were done with the Vpr deletion virus or virions transcomplemented with a Vpr expression plasmid in the producer cells. A sample of cells were harvested for total protein (see C) at this time. 48 hours post infection HIV-1 transcription was assayed for by measuring firefly luciferase and cell viability was monitored by the ATP assay CellTiter-glo (Promega). The data shown are the average mean values obtained in an experiment performed with quadruplicate samples and are representative of three independent experiments. (C and D) Western blots demonstrating loss of ZASC1 or ZBTB2 protein accumulation in (C) SUPT1 cells transfected with siRNAs targeting ZBTB2 or non-targeting (NT) siRNA or (D) ZASC1 and ZBTB2 knockout cells generated by CRISPR/CAS9 in SUPT1 and Jurkat T cell lines. (E) Co-immunoprecipitation assays of endogenous proteins. WT Jurkat, ZASC1 or ZBTB2 knockout cells (1X10^7^) were lysed and incubated with ZASC1, ZBTB2 or non-specific (NS) rabbit IgG. Co-immunoprecipitating complexes were detected by western blotting. (F) Cells (5X10^4^/well) were seeded in quadruplicate a 96 well plate, incubated for 48 hours and assayed for cell viability using the ATP assay CellTiter-glo (Promega). (G) Cells were passaged for the indicated time, counted, plotted and fitted to a growth curve using and the doubling time and 95% confidence interval was calculated using Prism graphing software. Error bars indicate the standard deviation of the data in all panels. ANOVA analysis was performed and for P-values < 0.05 a Tukey’s HSD was performed and relevant P-values reported.

Previously we showed that ZASC1 stimulates HIV-1 transcription elongation [38]. Since ZASC1 and ZBTB2 interact, our next goal was to determine if ZBTB2 affected HIV-1 transcription. To determine the effect of ZBTB2 on the HIV promoter, we performed transient transfection experiments in Jurkat T cells with the HIV-gLuc and tk-cLuc reporters. Overexpressing WT ZBTB2 repressed the basal, TAT-independent activity of the HIV-1 promoter by 7.1-fold (Fig 3A left graph) and repressed TAT-activated promoter activity by 22-fold (Fig 3A third graph). These data confirm that ZBTB2 is a repressor of the HIV-1 promoter. The POZ domains of many POK/ZBTB transcription factors are required for repression activity [49,50]. In contrast to WT ZBTB2, expressing a ΔPOZ ZBTB2 variant (deletion of a.a.1-79) reduced basal transcription by only 2.1-fold (Fig 3A left graph) and had no effect on TAT-activated transcription of the HIV-1 promoter (Fig 3A third graph), despite expression of the ΔPOZ ZBTB2 protein to WT levels (Fig 1C). Importantly, no repression was observed with the co-transfected control herpes simplex virus thymidine kinase promoter in either the absence (Fig 3A second graph) or presence of TAT (Fig 3A fourth graph). These data demonstrate that the ZBTB2 POZ domain is required for ZBTB2 repression of the HIV-1 promoter.

To determine if ZBTB2 affected HIV-1 gene expression in the context of viral infection, we performed siRNA-mediated ZBTB2 depletion in the SupT1 T-cell line and then challenged the siRNA treated cells with a VSV-G pseudotyped HIV-1 vector with nonsense mutations in the envelope and Vpr genes and firefly luciferase inserted into the Nef locus. As Vpr has been implicated in HIV-1 transcription, we took advantage of the fact that Vpr is a virion protein, and can be trans-complemented by co-transfecting a Vpr expression vector into virus-producing cells. ZBTB2 depletion had no effect on gene expression from an HIV-1 vector trans-complemented with Vpr (Fig 3B black bars). However, in stark contrast, ZBTB2-depleted cells showed a 3.9-fold increase in reporter gene expression (Fig 3B dark grey bars) when Vpr was not trans-complemented. A cellular viability assay that measures ATP levels (Cell titer glo, Promega, Madison WI) showed no differences between siZBTB2 and non-targeting siRNA control (Fig 3B light grey bars). Further, cells transfected with siRNAs against ZBTB2 showed undetectable levels of ZBTB2 by western blotting, but no effect on cellular lamin B (Fig 3C), a nuclear structural protein commonly used as a loading control. Thus, siRNA depletion of ZBTB2 suggests that, while ZASC1 is an activator, ZBTB2 is a repressor of HIV transcription in the absence of Vpr.

### CRISPR/Cas9 deletion of ZASC1 and ZBTB2

To further explore the roles of ZASC1 and ZBTB2 in HIV-1 transcription, we used CRISPR/Cas9-mediated, homology-directed repair (HDR) to knock out ZASC1 or ZBTB2 expression (Fig 3D) independently in both Jurkat and SupT1 T-cells, which have been extensively used in HIV-1 research [1,2]. Once we had obtained cell lines lacking ZASC1 and ZBTB2 expression, we asked if we could detect the interaction of the endogenous proteins. Anti-ZASC1 antibodies co-immunoprecipitated ZBTB2 from WT Jurkat cells, but not from the ΔZASC1 or ΔZBTB2 deletion cells (Fig 3E center panel). Similarly, anti-ZBTB2 antibodies co-immunoprecipitated ZASC1 from WT Jurkat cells but not from the ΔZASC1 or ΔZBTB2 cells (Fig 3E bottom panel). These results confirm the interaction of endogenous ZASC1 and ZBTB2 in cells.

Importantly, we observed no differences in cell viability between the cell lines as measured by the mitochondrial ATP CellTiter-glo assay (Fig 3F). Further, growth curve analysis revealed no differences between the parental cell lines and the ZASC1- or ZBTB2-deleted lines in growth rates (Fig 3G).

### Deletion of ZASC1 reduces HIV-1 gene expression and HIV-1 replication

**Fig 4:**
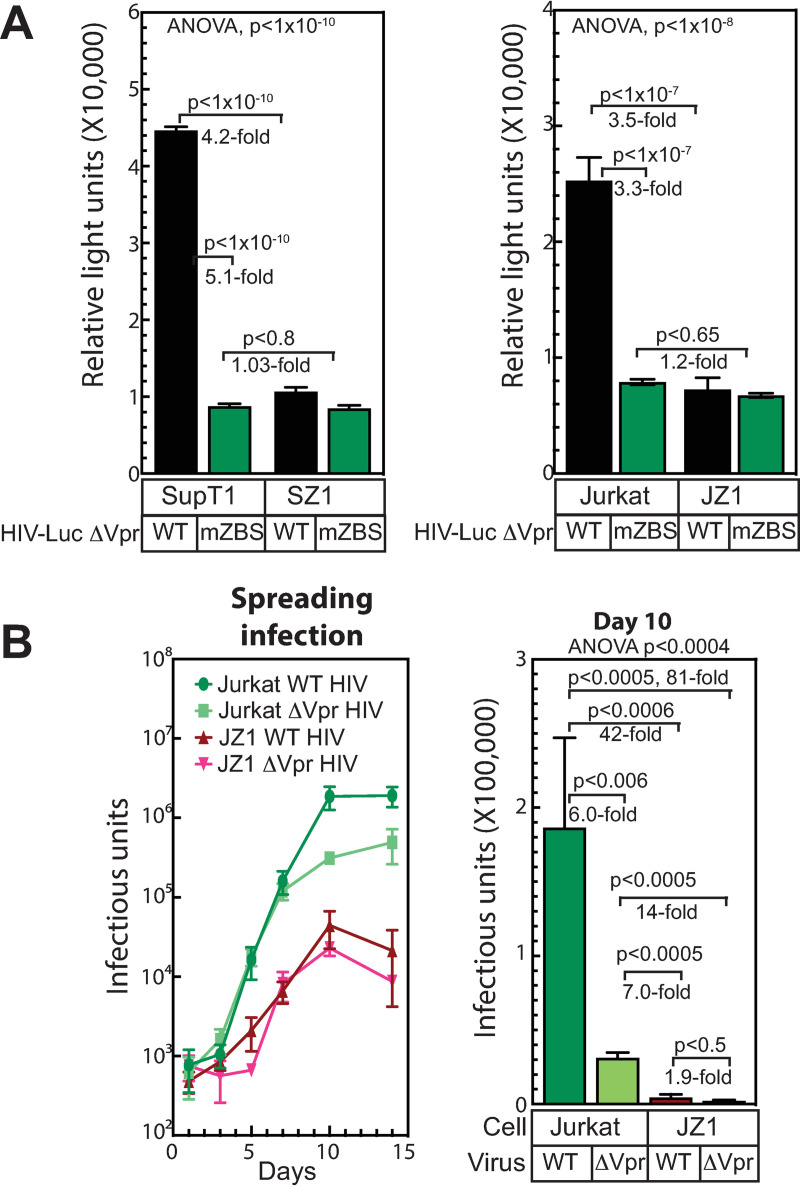
ZASC1 deletion reduces HIV-1 gene expression and viral replication. (A) WT or ΔZASC1 Jurkat and SupT1 cells were challenged with equivalent amounts (based on p24 levels) of either VSV-G pseudotyped HIV-1 vector with nonsense mutations in envelope and Vpr, luciferase in the NEF locus, and either a WT LTR or an LTR with all 4 ZASC1 binding sites mutated (mZBS) [38]. (B) Replication of WT HIV-1 or HIV-1 with a Vpr nonsense mutation (ΔVpr) in WT or ΔZASC1 Jurkat cells. Cells (1X10^6^) were infected with the indicated virus at an moi of 0.005. Supernatant was titered in quadruplicate on TZMBL reporter cells at the indicated time points and infectious units counted by X-gal staining. The data shown are the average mean values obtained in an experiment performed with quadruplicate samples and are representative of three independent experiments. The bar graph is a focus on the day 10 time point. Error bars indicate the standard deviation of the data in all panels. ANOVA analysis was performed and for P-values < 0.05 a Tukey’s HSD was performed and relevant P-values reported.

Similar to the effects we have previously reported for ZASC1 knockdown and ZASC1 dominant negative expression [38], CRISPR/Cas9 deletion of ZASC1 resulted in a 4.2-fold reduction in HIV-1 gene expression in SupT1 cells and 3.5-fold reduction in Jurkat cells (Fig 4A black bars) when challenged with virions of a VSV-G pseudotyped HIV-1 reporter vector with nonsense mutations in Env and Vpr, GFP-nanoluciferase (GFP-nLuc) inserted in the Nef locus and a WT LTR promoter. Importantly, when the WT and ΔZASC1 cell lines were challenged with virions of an HIV-1 vector in which all of the ZASC1 binding sites in the LTR promoter had been mutated [38], no significant reduction in gene expression was observed in SupT1 ΔZASC1 cells and Jurkat ΔZASC1 cells relative to WT cells (Fig 4A green bars). As previously reported, the mZBS promoter is significantly impaired in TAT-activation of transcription elongation [38] but retains clearly measurable basal activity, so this result Is not due to general promoter inactivation. In the experiments shown here, this mZBS virus showed a similar 5.1-fold and 3.3-fold reduction in reporter gene expression in WT SupT1 and Jurkat cells, respectively. Demonstrating that the effects of ZASC1 deletion on HIV-1 gene expression are mediated primarily through ZASC1 recruitment to specific binding sites in the HIV-1 promoter and are not off-target effects.

To determine the contribution of ZASC1 to viral replication, Jurkat and Jurkat ΔZASC1 cells were challenged with replication-competent WT HIV-1 (Fig 4B). Because of the link to Vpr we observed with ZBTB2 knockdown (Fig 3B) and the interaction of ZASC1 with ZBTB2 (Fig 1), we also challenged these cells with a replication competent HIV-1 with a nonsense mutation in Vpr. Cells (1X10^6^) were challenged at a low (0.005) multiplicity of infection and passaged for 14 days and viral release into the media was measured. Replication of WT and ΔVpr virus were indistinguishable in Jurkat cells for the first seven days, with ΔVpr virus ultimately yielding 4-fold less virus by day 10. In stark contrast, both viruses showed a severe replication defect in the ΔZASC1 cell line. By 10 days postinfection, WT virus exhibited a 42-fold reduction in virus production relative to the WT cells, while the ΔVpr virus was reduced 14-fold relative to the ΔVpr replication in Jurkat cells. Taken together, these data suggest the reduction in HIV-1 gene expression upon ZASC1 deletion leads to a significant decrease in viral replication.

### Deletion of ZBTB2 enhances HIV-1 gene expression and HIV-1 replication in VPR deletion strains

**Fig 5:**
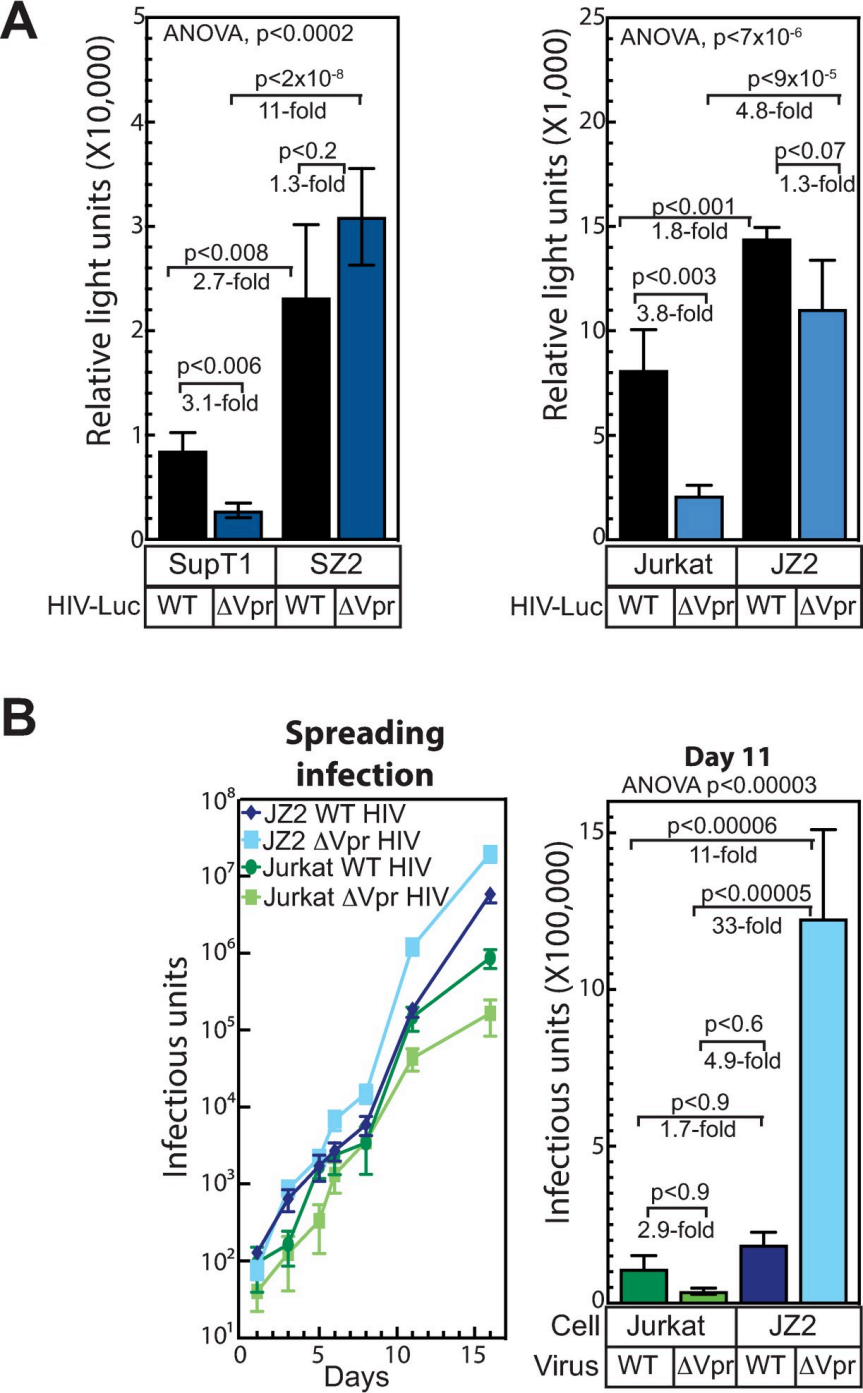
ZBTB2 deletion enhances HIV-1 gene expression and viral replication of VPR knockout strains. (A) WT or ΔZBTB2 Jurkat and SupT1 cells were challenged with equivalent amounts (based on p24 levels) of either VSV-G pseudotyped HIV-1 vector with nonsense mutations in envelope, luciferase in the NEF locus, and either a WT Vpr or a Vpr nonsense mutant (ΔVpr). (B) Replication of WT or ΔVpr HIV-1 in WT or ΔZBTB2 Jurkat cells. Cells (1X10^6^) were infected with the indicated virus at an moi of 0.005. Supernatant was titered in quadruplicate on TZMBL reporter cells at the indicated time points and infectious units counted by X-gal staining. The data shown are the average mean values obtained in an experiment performed with quadruplicate samples and are representative of three independent experiments. The bar graph is a focus on the day 11 time point. Error bars indicate the standard deviation of the data in all panels. ANOVA analysis was performed and for P-values < 0.05 a Tukey’s HSD was performed and relevant P-values reported.

To determine the effects of ZBTB2 on HIV-1 gene expression, we challenged the ZBTB2 CRISPR/Cas9 deletion cell lines with virions of selected HIV-1 reporter vectors. Challenge with a VSV-G-pseudotyped HIV-1 reporter vector with a WT *vpr* gene resulted in a 2.7-fold increase in HIV-1 gene expression in SupT1 ΔZBTB2 cells and 1.8-fold increase in Jurkat ΔZBTB2 cells relative to the corresponding WT Jurkat cells (Fig 5A black bars). This suggests that while Vpr may counteract ZBTB2, completely lacking ZBTB2 may be more efficient for HIV-1 gene expression. In striking contrast, when challenged with an HIV-1 vector that was deleted for Vpr, SupT1 ΔZBTB2 cells exhibited an 11-fold increase, and Jurkat ΔZBTB2 cells a 4.8-fold increase in HIV-1 gene expression (Fig 5A blue bars). Given that deletion of Vpr reduced HIV-1 reporter expression in the WT SupT1 and Jurkat cells (3.1 and 3.8-fold, respectively), deletion of ZBTB2 restored the gene expression of the ΔVpr virus to near WT levels. Taken together with the siRNA data with a Vpr deletion virus (Fig 3B), these data demonstrate that ZBTB2 is a repressor of HIV-1 gene expression and that Vpr antagonizes this repression.

To determine the contribution of ZBTB2 to viral replication and the contribution of Vpr to this effect, Jurkat and Jurkat ΔZBTB2 cells were challenged with replication-competent WT and ΔVpr HIV-1 (Fig 5B). Cells (1X10^6^) were challenged at a low (0.005) multiplicity of infection and passaged for 16 days and viral release into the media was measured. As we observed above (Fig 4B), WT and ΔVpr virus replicated similarly through day 8 in the time course. By day 11, the ΔVpr virus showed a 2.9-fold reduction in viral replication relative to WT virus replication in WT Jurkat cells. In contrast, in Jurkat ΔZBTB2 cells, the ΔVpr virus showed a 6.6 fold increase in replication relative to WT virus. This was a 33-fold increase in the ΔVpr virus replication in ΔZBTB2 vs WT cells and 11-fold greater replication than WT virus replication in WT cells. Interestingly, WT virus showed only a modest 1.7-fold increase in replication in ΔZBTB2 vs WT cells, suggesting that while Vpr counteracts negative effects of ZBTB2, some effects of Vpr’s other functions, such as cell cycle arrest or apoptosis, exert negative consequences on HIV-1 replication in the absence of ZBTB2. Thus, our data indicate that ZBTB2 is a repressor of HIV-1 transcription and virion-associated Vpr overcomes this repression and enhances HIV-1 replication.

### ZASC1 activates the HIV-1 promoter

**Fig 6:**
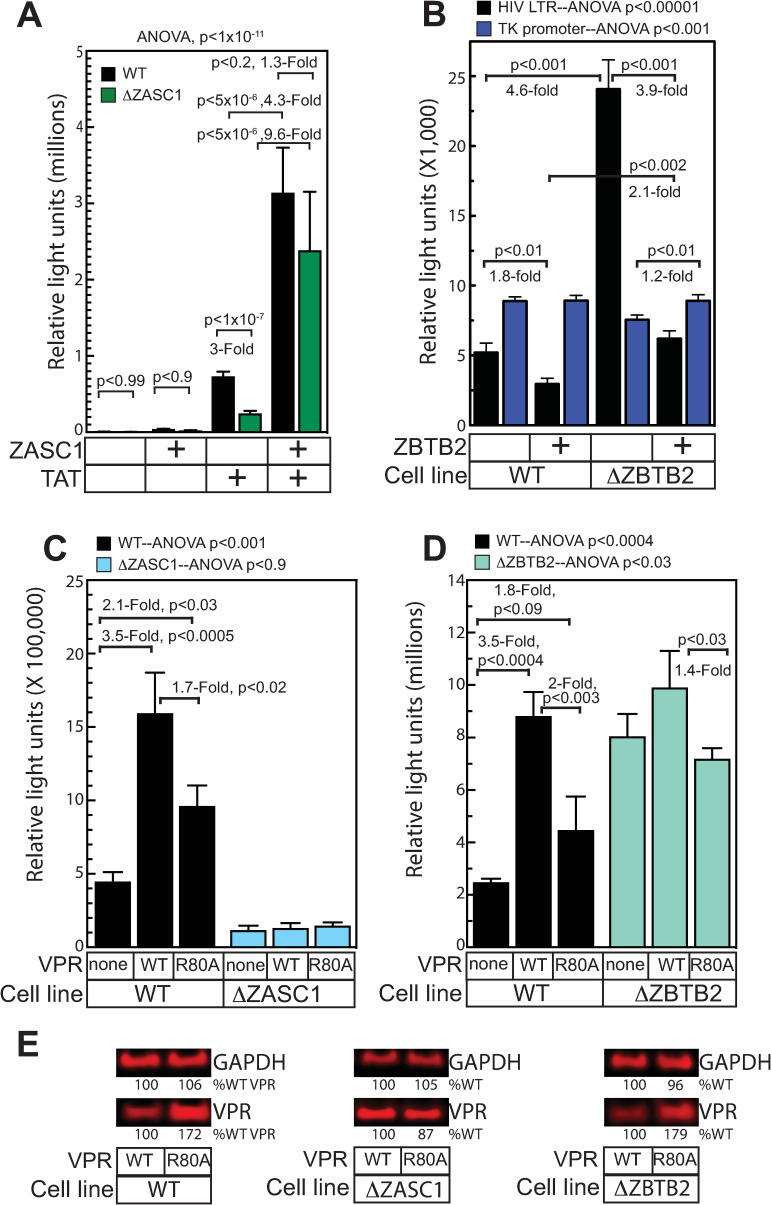
ZASC1, ZBTB2 and Vpr regulate HIV-1 LTR promoter activity. (A) WT Jurkat and ΔZASC1 Jurkat cells transfected with reporter plasmids expressing *Gaussia* luciferase from the HIV-1 promoter and with the ZASC1 and/or TAT expression plasmids indicated in the table at the bottom. Raw relative light units of HIV-1 LTR promoter-driven reporter gene expression for each cell line was reported in the presence and absence of ZASC1 and TAT. (B) WT and ΔZBTB2 Jurkat cells were transfected with reporter plasmids containing HIV-1 promoter-driven *Gaussia* luciferase and HSV TK-promoter driving *Cypridinia* luciferase expression. Raw luciferase activity for both the HIV-1 LTR and HSV TK promoters is plotted. (C) WT and ΔZASC1 or (D) ΔZBTB2 Jurkat cells were transfected with the above luciferase reporter plasmids, TAT, and plasmids expressing either WT Vpr or the cell-cycle deficient Vpr R80A mutant. Raw *Gaussia* luciferase activity for the HIV-1 LTR promoter divided by the raw *Cypridinia* luciferase activity of the TK promoter is plotted as relative light units. The data shown in each of panels are the average mean values obtained in an experiment performed with quadruplicate samples and are representative of three independent experiments. (E) Western blots showing expression of WT and R80A Vpr mutants in each cell line. Numbers indicate band intensity relative to WT Vpr lanes. Error bars indicate the standard deviation of the data in all panels. ANOVA analysis was performed and for P-values < 0.05 a Tukey’s HSD was performed and relevant P-values reported.

To confirm the effects of ZASC1 on the HIV-1 promoter outside of infection and to confirm that the phenotype of our knockout cells is primarily due to deletion of ZASC1, we transiently transfected Jurkat and Jurkat ΔZASC1 cells with a plasmid containing the HIV-1 LTR promoter driving expression of *Gaussia* luciferase (HIV-gLuc). A control plasmid with the herpes simplex virus (HSV) thymidine kinase (TK) promoter driving *Cypridina* luciferase (tk-cLuc) was included as a transfection efficiency control. We previously reported that ZASC1 expression had limited effects on the basal activity of the HIV-1 promoter, but stimulated activation of the promoter by the HIV-1 TAT protein [38]. Consistent with this, in the absence of Tat, expressing ZASC1 in both Jurkat and Jurkat ΔZASC1 cells moderately stimulated the HIV-1 promoter approximately 6-fold in both cell lines (Fig 6A). However, TAT activation in the Jurkat ΔZASC1 cell line was significantly impaired, stimulating the HIV-1 promoter 3-fold less than WT Jurkat cells. Co-expressing ZASC1 and TAT further stimulated the HIV-1 LTR promoter in both cell lines, and brought the HIV-1 promoter in the Jurkat ΔZASC1 cells nearly to the level seen in WT Jurkat cells (1.3-fold less, but not significantly different). These data are consistent with the previously inferred ZASC1 role of stimulating HIV-1 TAT mediated transcription elongation and demonstrate that the primary defect to HIV-1 transcription in the ΔZASC1 cell line is due to loss of ZASC1.

### ZBTB2 represses the HIV-1 promoter

We showed above (Fig 3A) that ZBTB2 represses both the basal and TAT-stimulated activity of the HIV-1 promoter but not the HSV tk promoter. To confirm that the phenotype of our ZBTB2 knockout cells is primarily due to effects of ZBTB2 deletion on the HIV-1 promoter, we transiently transfected Jurkat and Jurkat ΔZBTB2 cells with the HIV-gLuc and TK-cLuc reporter plasmids. (Fig 6B). Strikingly, the basal activity of the HIV-1 promoter was 4.6-fold higher in the Jurkat ΔZBTB2 cells than in the parental Jurkat cells. However, expression from the TK promoter was actually slightly (15%) lower in the Jurkat ΔZBTB2 cell line. Thus, ZBTB2 represses the HIV-1 promoter in WT Jurkat cells and loss of ZBTB2 increases basal activity of the HIV-1 but not the TK promoter. Expressing ZBTB2 in Jurkat ΔZBTB2 cells reduces HIV-1 promoter activity by 3.9-fold, to levels similar to WT Jurkat cells, while, under these conditions, ZBTB2 expression in WT Jurkat cells represses expression another 2-fold. Importantly, the TK promoter was unaffected by ZBTB2 expression. Taken together, these data indicate that ZBTB2 is a repressor of the HIV-1 promoter and that the increased expression observed in Jurkat ΔZBTB2 cells is primarily due to the loss of ZBTB2.

### Vpr stimulates the HIV-1 promoter through ZASC1 and ZBTB2

As shown above (Figs 3B and 5), HIV-1 Vpr blocks the repressive effects of ZBTB2 on the HIV-1 promoter. Vpr has been reported to stimulate HIV-1 transcription by inducing G2 cell cycle arrest where the HIV-1 promoter is more active for poorly understood reasons [6–8]. To determine if ZASC1 and ZBTB2 contribute to the activation of the HIV-1 promoter by Vpr, we transfected either the ΔZASC1 (Fig 6C) or the ΔZBTB2 (Fig 6D) cell lines with the HIV-1 and TK reporter plasmids along with plasmids expressing either WT Vpr or the cell-cycle arrest-defective mutant Vpr R80A [12]. Transfecting WT Vpr activated the HIV-1 promoter 3.5-fold (Fig 6C and 6D) in WT cells, while the Vpr R80A mutant only activated the HIV-1 promoter 2.1 and 1.8-fold (Fig 6C and 6D, respectively). While the R80A mutation also has been implicated in affecting other Vpr functions, namely interaction with SLX4 binding and MUS81 depletion, taken together [18,25], these data suggest that the Vpr transcription activation effect depends at least in part on effects linked to Vpr-mediated cell-cycle arrest. In addition, the R80A mutant expressed as well or better than WT Vpr in these cell lines (Fig 6E), so lack of R80A expression cannot explain the reduced transcription activity. Importantly, neither WT nor R80A Vpr had any significant effect on HIV-1 expression in the ΔZASC1 (Fig 6C) or ΔZBTB2 (Fig 6D) cell lines, implying that Vpr effects on HIV-1 transcription depend on ZASC1 and ZBTB2.

### ZASC1 and ZBTB2 do not affect HIV-1 2LTR circle formation or cDNA integration

To further verify that ZASC1 and ZBTB2 primarily affect HIV-1 gene expression and not other steps in HIV-1 infection, we took two approaches that do not rely on HIV-1 transcription. First, we analyzed the formation of the reverse transcription/integration byproduct 2LTR circles (S2A Fig). Two LTR circle formation is a widely accepted measure of HIV-1 infection, reverse transcription and nuclear import [58]. We infected the Jurkat WT, ΔZASC1 and ΔZBTB2 cell lines with replication competent HIV-1 WT and ΔVpr and 48 hours post infection, harvested the total DNA from the cells and assayed for 2LTR circles and a cellular PDBG1 gene as control for cell number [58]. For HIV-1 WT virus, variations in 2LTR circle formation between WT cells and either ΔZASC1 or ΔZBTB2 cells were not statistically significant. A 47% difference in 2LTR formation between ΔZASC1 and ΔZBTB2 cell lines was statistically significant but is small compared to the effects on LTR promoter transcription discussed above. Further, for HIV-1 ΔVpr virus no differences in 2LTR circle formation were observed between any of the cell lines (S2A Fig). We also observed only minor differences (up to 26%) in integrated viral DNA, as determined by real-time QPCR that recognized the HIV-1 promoter (-54 to +32), when the ΔZASC1 and ΔZBTB2 cell lines were infected with a ΔVpr vector and passaged for 7 days to deplete unintegrated DNA (S2B Fig). Unfortunately, equivalent passaging to deplete unintegrated cDNA cannot be performed to analyze cDNA integration for a Vpr-positive virus, since Vpr arrests the cell cycle. Nevertheless, in addition to the 2LTR circle results it should be noted that in ΔZASC1 cells the WT and ΔVpr HIV-1 productive replication phenotypes are indistinguishable (Fig 4B) and that in ΔZBTB2 cells the LTR transcription phenotype is primarily observed with ΔVpr virus (Fig 5A and 5B), whose proviral integration is analyzed in S2B Fig. Together, these data support the model that the effects of ZASC1 and ZBTB2 on LTR promoter-directed gene expression are not a secondary consequence of effects on other facets of HIV-1 replication.

#### ZBTB2—SP1 interaction is weak relative to ZBTB2—ZASC1 interaction

ZBTB2 has been reported to interact with the cellular transcription factor SP1, which is also critical for HIV-1 gene expression [2,51,52,59]. Fig 2 shows that ZASC1 interacting with ZBTB2 causes nuclear relocalization of ZBTB2, while SP1 expression has no effect on ZBTB2 localization (Fig 2H vs 2L). To compare the interactions between ZBTB2 and either ZASC1 or SP1, HEK293 cells were transfected with expression plasmids encoding epitope-tagged variants of the three proteins and immunoprecipitated with anti-Flag beads. While ZBTB2 strongly co-immunoprecipitated with ZASC1 (Figs 1B and 1C, and S3A), it only weakly co-immunoprecipitated with SP1, at the limits of detection upon long exposure (S3A Fig). Additionally, Flag-ZASC1 co-immunoprecipitated Myc-ZBTB2 but not Myc-Sp1 (S3A Fig). Thus, under these conditions, the complex of ZBTB2 with ZASC1 is significantly more robust than the ZBTB2—SP1 interaction.

#### ZBTB2 does not detectably interact with Vpr

As shown above, Vpr antagonizes ZBTB2 repression of the HIV-1 promoter (Figs 3B, 5, 6C and 6D). To determine if Vpr may be directly binding ZBTB2 and interfering with ZBTB2 function, we transfected HEK293 cells with expression plasmids encoding a Myc-tagged variant of ZBTB2 and Flag-tagged variants of either ZASC1 as a positive control or Vpr, and immunoprecipitated with anti-Flag beads. While ZBTB2 strongly immunoprecipitated with ZASC1, no ZBTB2 co-immunoprecipitation was observed with Vpr (S3B Fig). These data imply that, however Vpr affects ZBTB2 function, it is likely not through direct interaction.

#### ZBTB2’s POZ domain interacts with cellular HDACs

**Fig 7:**
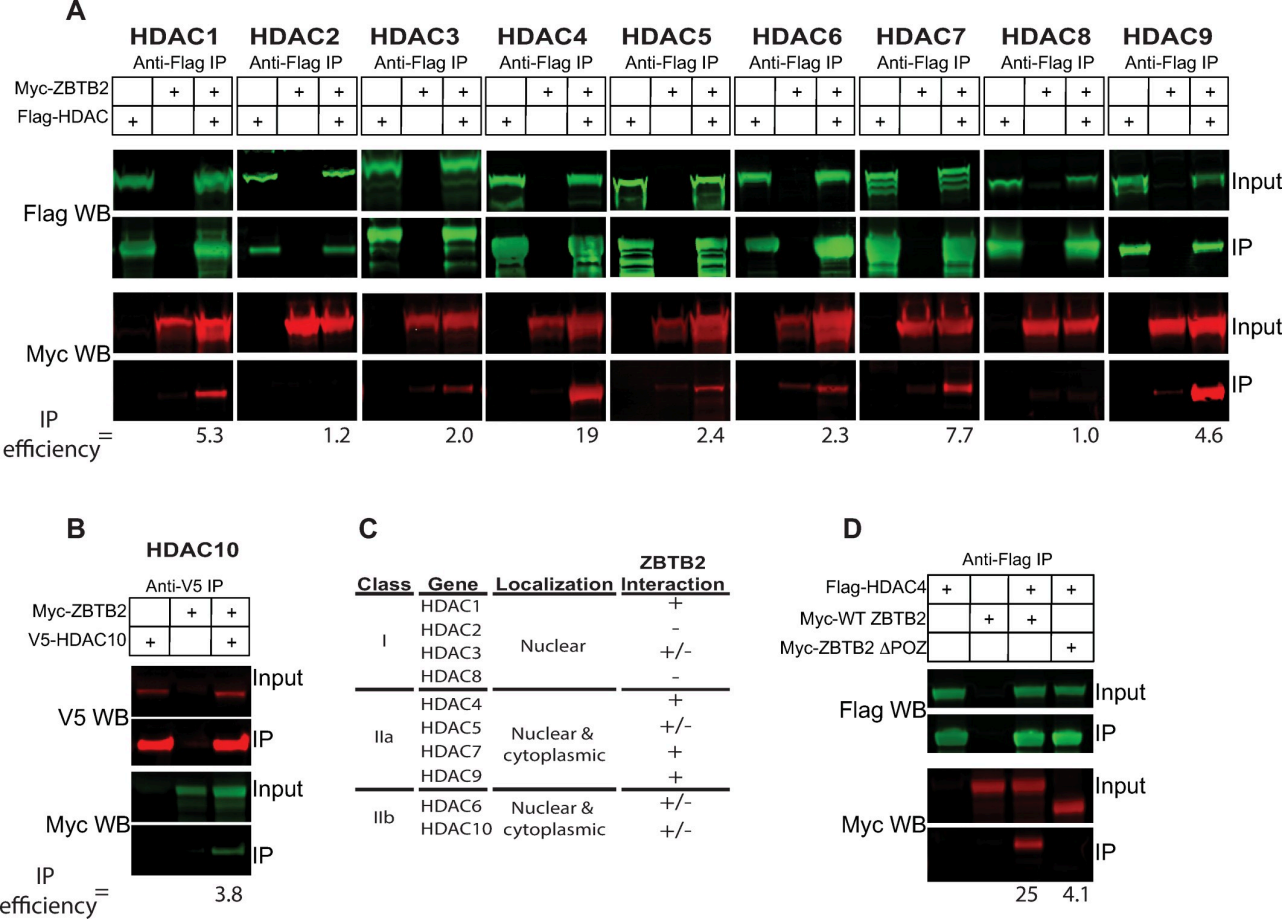
ZBTB2 POZ domain interacts with HDACs. HEK293 cells (1X10^7^) were transfected with expression plasmids encoding the epitope tagged forms of the indicated proteins. 48 h post-transfection, cells were lysed and epitope tagged proteins were immunoprecipitated (IP) with anti-Flag beads, separated by SDS-PAGE and analyzed by western blotting (WB) using the indicated antibodies as described in Materials and Methods. (A) Co-immunoprecipitation of Myc-ZBTB2 and Flag-tagged HDACs 1–9 or (B) V5 tagged HDAC10. (C) Summary of ZBTB2 and cellular HDAC interactions. Interaction (+) was defined as having an IP efficiency greater than 4.5, weak interaction (+/-) as less than 4.5 and no interaction (-) as less than 1.5. (D) Co-immunoprecipitation assays of Flag-HDAC4 and Myc-WT ZBTB2 or Myc-ΔPOZ ZBTB2. IP efficiency is the ratio of % starting material immunoprecipitated by HDAC verses the % starting material precipitated with no HDAC (e.g. ratio of lane 3:lane 2 in A and B).

ZBTB2 is a member of the POK (POZ and Kruppel) family of transcription factors. These proteins share an N-terminal POZ domain and C-terminal C2H2 zinc fingers (Fig 1A). POZ domains frequently interact with cellular histone deacetylase (HDAC) complexes [49]. Since our data show that ZBTB2 represses HIV-1 gene expression, and that the POZ domain (a.a. 1–79) is essential for this repression (Fig 3A), we investigated if ZBTB2 interacts with any of the ten class I and class II cellular HDACs. HEK293 cells were transfected with expression plasmids encoding Flag-tagged HDACs 1 to 9 (Fig 7A) or V5-tagged HDAC10 (Fig 7B) and Myc-tagged ZBTB2. Immunoprecipitation efficiency was calculated by determining the ratio of the percentage of starting Myc-ZBTB2 precipitated in the presence and absence of the HDAC protein. This assay showed that HDACs 1, 4, 7 and 9 were strong ZBTB2 interactors (defined as immunoprecipitation efficiency ≥ 4.5), with HDAC4 showing the most robust interaction (Fig 7A and 7B). HDAC 3, 5, 6 and 10 immunoprecipitated ZBTB2 detectably but more weakly (Fig 7A, 7B and 7C). To determine if this interaction was mediated by the ZBTB2 POZ domain, HEK293 cells were transfected with expression plasmids encoding Flag-tagged HDAC4 and Myc-tagged ZBTB2 deleted for the first 79 amino acids (ZBTB2 ΔPOZ). Despite good expression, ZBTB2 ΔPOZ did not efficiently co-immunoprecipitate with Flag-HDAC4 (Fig 7D). These data indicate that the ZBTB2 POZ domain interacts with cellular HDAC4.

#### ZASC1 recruits ZBTB2 to the HIV-1 promoter

**Fig 8:**
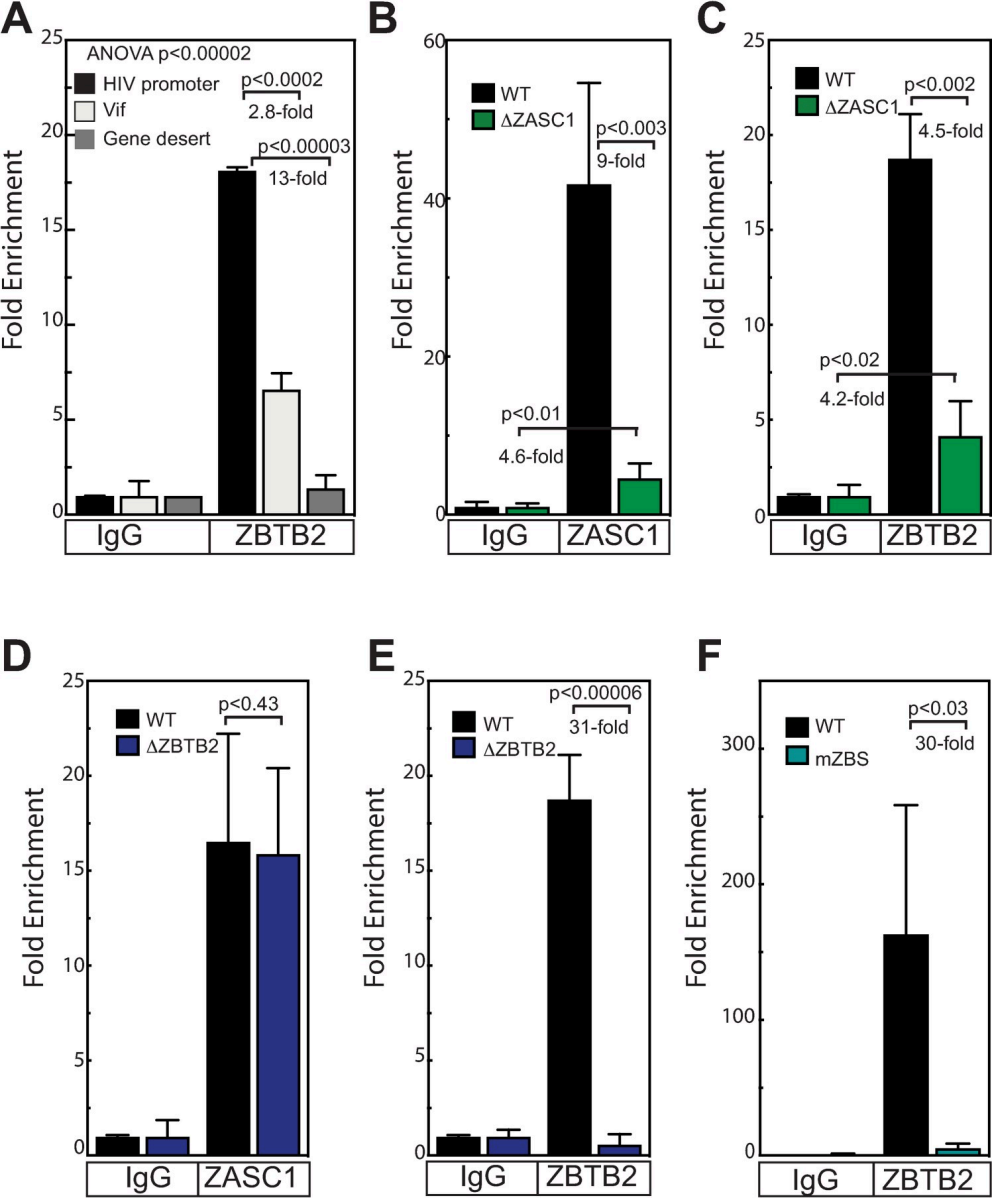
ZASC1 regulates recruitment of ZBTB2 to the HIV-1 promoter. (A) ChIP results from Jurkat cells transduced with NL43E-R-Luc using an antibody against ZBTB2 or non-specific IgG control antibody and primer sets targeting the HIV-1 promoter (-54 to +32) and downstream sequences in the Vif gene (+4,599 to +4,619) or a region on chromosome 12 that lacks active genes (gene desert). ChIP analysis of (B) ZASC1 and (C) ZBTB2 recruitment to the HIV-1 promoter in infected WT Jurkat and Jurkat ZASC1 knockout cells. (D-E) ChIP analysis of (D) ZASC1 and (E) ZBTB2 recruitment to the HIV-1 promoter in infected WT Jurkat and Jurkat ZBTB2 knockout cells. (F) ChIP analysis of ZBTB2 recruitment to the HIV-1 promoter in WT Jurkat cells infected with an HIV-1 vector with either a WT LTR or an LTR variant with all four ZASC1 binding sites mutated. Immunoprecipitation and Real-time PCR analysis were performed and normalized to input controls as a percentage of starting material for immunoprecipitation and fold enrichment of experimental IPs relative to IgG controls reported. For each panel, the data shown are the average mean values obtained in an experiment performed with quadruplicate samples and are representative of three or more independent experiments. For (A), ANOVA analysis was performed and for P-values < 0.05 a Tukey’s HSD was performed and relevant P-values reported. For (B to F) P-values were calculated using a standard Student’s t-test and significant changes are indicated.

Previously we showed that ZASC1 binds specific sequences in the HIV-1 promotor [38]. To determine if ZBTB2 is present at the HIV-1 promoter and explore how the ZASC1 and ZBTB2 interaction regulates HIV-1 transcription, we performed Chromatin Immuno-Precipitation (ChIP) assays and used quantitative real time PCR to determine the amount of selected DNA loci precipitated (ChIP-QPCR). Jurkat cells were infected with NL43E-R-Luc, an HIV-1 vector with nonsense mutations in the envelope and Vpr genes and firefly luciferase (fLuc) inserted into the Nef locus. ChIP-QPCR was performed with anti-ZBTB2 antibodies. Primer/probe sets were designed that recognized the HIV-1 promoter (-54 to +32) and downstream sequences in the Vif gene (+4,599 to +4,619). To control for the fact that each integrated provirus contains two LTR target sites but only one Vif gene, we used a full-length proviral clone—containing both LTRs and one Vif gene—to generate the QPCR standard curves for both primer sets. This approach normalized for any amplification differences between different primer/probe sets due to copy number differences in the proviral target sequences. As shown in Fig 8A, relative to non-specific IgG control pulldowns, the ZBTB2 immunoprecipitate was enriched 18-fold for the HIV-1 promoter sequences, but only enriched 6.6-fold for the downstream Vif sites. These findings are greater than the 2-fold copy number difference and consistent with ZBTB2 binding primarily at DNA sequences in the HIV-1 promoter. As a further specificity control, a primer/probe set to a gene-poor region of chromosome 12 [60] was amplified in parallel with a primer/probe set to the HIV-1 promoter. This gene desert sequence showed minimal enrichment in ZBTB2 immunoprecipitates (1.4-fold) relative to non-specific IgG control pulldowns and represented recovery of 25-fold less total DNA than for the HIV-1 promoter. These data, in conjunction with our previous results [38], confirm that both ZASC1 and ZBTB2 are specifically recruited to the HIV-1 promoter.

To determine if the interaction between ZASC1 and ZBTB2 affect either’s recruitment to the HIV-1 promoter, we performed ChIP experiments in Jurkat cell lines deleted for either ZASC1 or ZBTB2 by CRISPR/Cas9. Anti-ZASC1 antibody showed a 41-fold enrichment signal at the HIV-1 promoter in WT Jurkat cells but only a slight residual enrichment signal (on average, a 2 to 4-fold) in ΔZASC1 cells (Fig 8B). This suggests that there is some degree of non-specific binding of the ZASC1 antibody to other factors at the HIV-1 promoter, but this was always at least 9-fold below what was observed in ZASC1-positive WT cells. ZBTB2 was enriched 19-fold at the HIV promoter in WT cells, while ΔZASC1 cells showed only a 4.2-fold enrichment in ZBTB2 (Fig 8C) recruitment, showing that ZASC1 contributes strongly to ZBTB2 recruitment. In contrast, ΔZBTB2 cells largely retained ZASC1 at the promoter (Fig 8D; P<0.43), but lost ZBTB2 recruitment (Fig 8E). Taken together, these data show that ZASC1 markedly enhances the recruitment of ZBTB2 to the HIV-1 promoter. In support of this conclusion, Jurkat cells infected with an HIV-1 vector lacking ZASC1 binding sites in the LTR promoter also show a loss of ZBTB2 recruitment (Fig 8F). While other factors, such as ZBTB2 interaction with SP1 and ZBTB2 DNA binding [51] may also contribute, our data implies that ZASC1 is the primary factor targeting ZBTB2 to the HIV promoter.

#### ZBTB2 represses the HIV-1 promoter by altering histone acetylation

**Fig 9:**
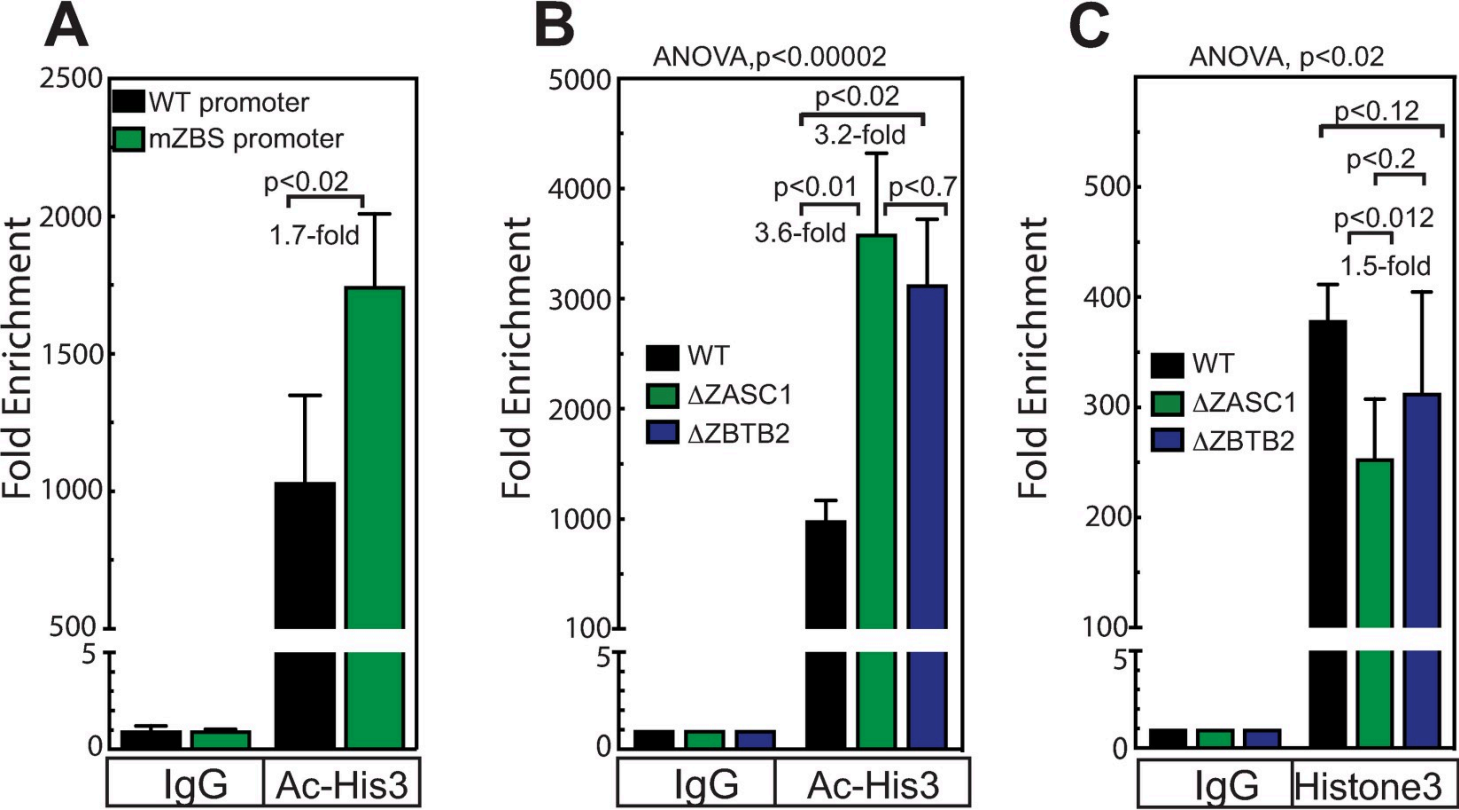
ZBTB2 regulates histone acetylation at the HIV-1 promoter. ChIP analysis of (A) histone H3 acetylation for the HIV-1 promoter in WT Jurkat cells infected with an HIV-1 vector with either a WT LTR or an LTR variant with all four ZASC1 binding sites mutated. ChIP analysis of (B) histone H3 acetylation and (C) total histone H3 on the HIV-1 promoter in HIV-1 vector infected WT Jurkat, Jurkat ZASC1 knockout cells and Jurkat ZBTB2 knockout cells. Immunoprecipitation and Real-time PCR analysis were performed and normalized to input controls as a percentage of starting material for immunoprecipitation and fold enrichment of experimental IPs relative to IgG controls reported. For each panel, the data shown are the average mean values obtained in an experiment performed with quadruplicate samples and are representative of three or more independent experiments. Error bars indicate the standard deviation of the data in all panels. For (A), P-values were calculated using a standard Student’s t-test and significant changes are indicated, for (B & C) ANOVA analysis was performed and for P-values < 0.05 a Tukey’s HSD was performed and relevant P-values reported.

Since the POZ domain was essential for both the ZBTB2 repressive activity (Fig 3A) and for recruiting HDAC4 (Fig 7A and 7D), we examined the effect of ZBTB2 on histone H3 acetylation at the HIV-1 promoter. ChIP assays were performed under conditions that do or do not support ZASC1-mediated recruitment of ZBTB2 to the HIV-1 promoter, as shown in the section above. Infection with an HIV-1 vector lacking ZASC1 binding sites in the LTR promoter resulted in a statistically significant increased enrichment of acetylated histones from 1000-fold to 1750-fold increase in histone acetylation at the promoter relative to a vector with a WT promoter (Fig 9A). Similarly, infection of either the ΔZASC1 or the ΔZBTB2 cell lines also showed substantial increases in acetylated histone H3 enrichments at the HIV-1 promoter (3,500-fold and 3,100-fold, respectively) relative to the enrichment in WT cells (990-fold)(Fig 9B). Consistent with nucleosomal remodeling at the HIV-1 promoter that occurs after histone acetylation which results in loss of a nucleosome [61–63], total histone H3 on the promoter was modestly reduced from 910-fold enrichment with WT to 460-fold and 660-fold, respectively, in the ΔZASC1 and ΔZBTB2 cell lines cells (Fig 9C), although only the reduction in the ΔZASC1 cells was statistically significant. These data, along with the failure to observe any alterations in 2LTR circle or integrated provirus levels with either the ΔZASC1 and ΔZBTB2 cell lines (S2 Fig), show that ZBTB2 is recruited by ZASC1, represses HIV-1 transcription by recruiting cellular HDACs that de-acetylate histones on the HIV-1 promoter, and also may have a very modest contribution to nucleosomal remodeling.

#### ZBTB2 localization is regulated by ZASC1 and the Vpr/ATR/DDR pathway

The above results show that regulation of ZASC1 interactions switches ZASC1 from an activator of HIV-1 transcription to a recruiter of the repressive ZBTB2 complex. Several lines of evidence suggested that this regulation may be due to activating the DNA damage response (DDR). First, our observation that Vpr impaired ZBTB2 repression of the HIV-1 promoter (Figs 3B and 5) implies that Vpr regulates ZBTB2 function. In addition, we observed that Vpr activation of the HIV-1 LTR was not observed in ΔZASC1 and ΔZBTB2 cell lines (Fig 6C and 6D). Further, HIV-1 Vpr causes cell cycle arrest by activating the ATR pathway to induce the DDR [19,20], and ZBTB2 has been implicated in regulating genes in the DNA damage pathway [51]. Finally, the HIV-1 LTR promoter is significantly more active in G2 arrested cells [6], which is consistent with our observation that the Vpr R80A mutant that is deficient in cell-cycle arrest activity is impaired in stimulating the HIV-1 promoter (Fig 6C and 6D). Given this, we explored if Vpr and DDR activation affect ZBTB2 function.

**Fig 10:**
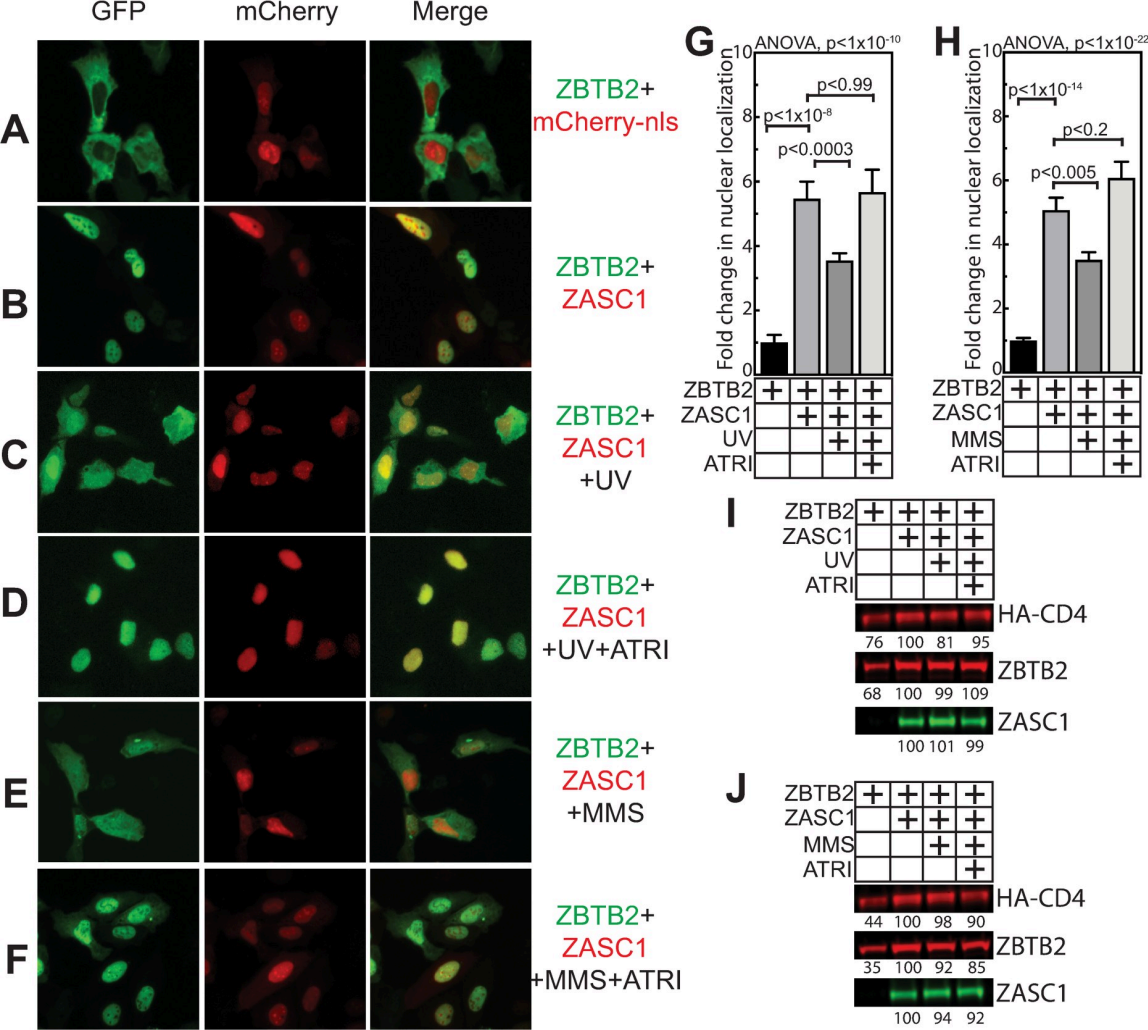
ZASC1 and the ATR kinase pathway regulate ZBTB2 localization. U2OS cells were transfected with GFP-ZBTB2 and either mCherry with a nuclear localization signal (A) or mCherry-ZASC1 (B-F) and imaged 24 hours post transfection. ATR kinase was activated by 320 nm UV light (C & D), 8 mM of the DNA damaging agent methyl methanesulfonate (MMS) ATR activation was inhibited by treating with 10 μM of ATR inhibitor (CAS 905973-89-9) (D, F). (G and H) To quantify the effects observed in (A-F), independent transfections were performed with the indicated conditions and the relative ZBTB2 fluorescence signals in the nucleus and a nucleus-adjacent cytoplasmic ring were measured in ≥100 cells, and the fold change in nuclear to cytoplasmic signal reported. (G) presents the effects of DNA damaging UV treatment and ATR inhibitor, and (H) presents the effects of 8 mM of the DNA damaging agent MMS and 10 μM ATR inhibitor. Error bars indicate the standard deviation of the data in all panels. ANOVA analysis was performed and for P-values < 0.05 a Tukey’s HSD was performed and relevant P-values reported. Western blots showing the effects of (I) UV or (J) MMS treatment on the levels of ZASC1, ZBTB2 and a HA-tagged CD4 control protein. Numbers indicate band intensity relative to lane expressing both ZASC1 and ZBTB2. Results are representative of three independent experiments.

To explore the effects of Vpr and the DDR on ZBTB2 function, we took advantage of our observations that a GFP-ZBTB2 fusion protein is primarily cytoplasmic (Figs 2F and 10A) but dramatically moves into the nucleus when co-expressed with mCherry-ZASC1 (Figs 2H and 10B). To explore the effect of DNA damage on ZASC1-mediated ZBTB2 localization, cells transfected with both mCherry-ZASC1 and GFP-ZBTB2 were exposed to DNA damaging 320 nm UV light for 5 minutes and then imaged 4 hours later. Strikingly, even in the presence of ZASC1, the UV treatment significantly increased ZBTB2 cytoplasmic localization at the expense of nuclear localization (Fig 10C and 10G). This relocalization from nucleus to cytoplasm could be blocked by treating with an ATR kinase inhibitor (Fig 10D and 10G). Multiple UV treatments and times post treatment were tested, and 5 min. exposure and imaging 4 hours post treatment were selected as resulting in maximal relocalization with the least amount of apoptosis. Consistently, the chemical DNA damaging agent methyl methanesulfonate (MMS), also caused cytoplasmic relocalization of ZBTB2 (Fig 10E and 10H), which was blocked with ATR kinase inhibitor treatment (Fig 10F and 10H). Importantly, these treatments had only minor effects on the protein levels of ZASC1, ZBTB2 or a control protein (co-transfected HA-tagged CD4) (Fig 10I and 10J), and even these minor variations did not correlate with the ZBTB2 relocalization phenotype.

**Fig 11:**
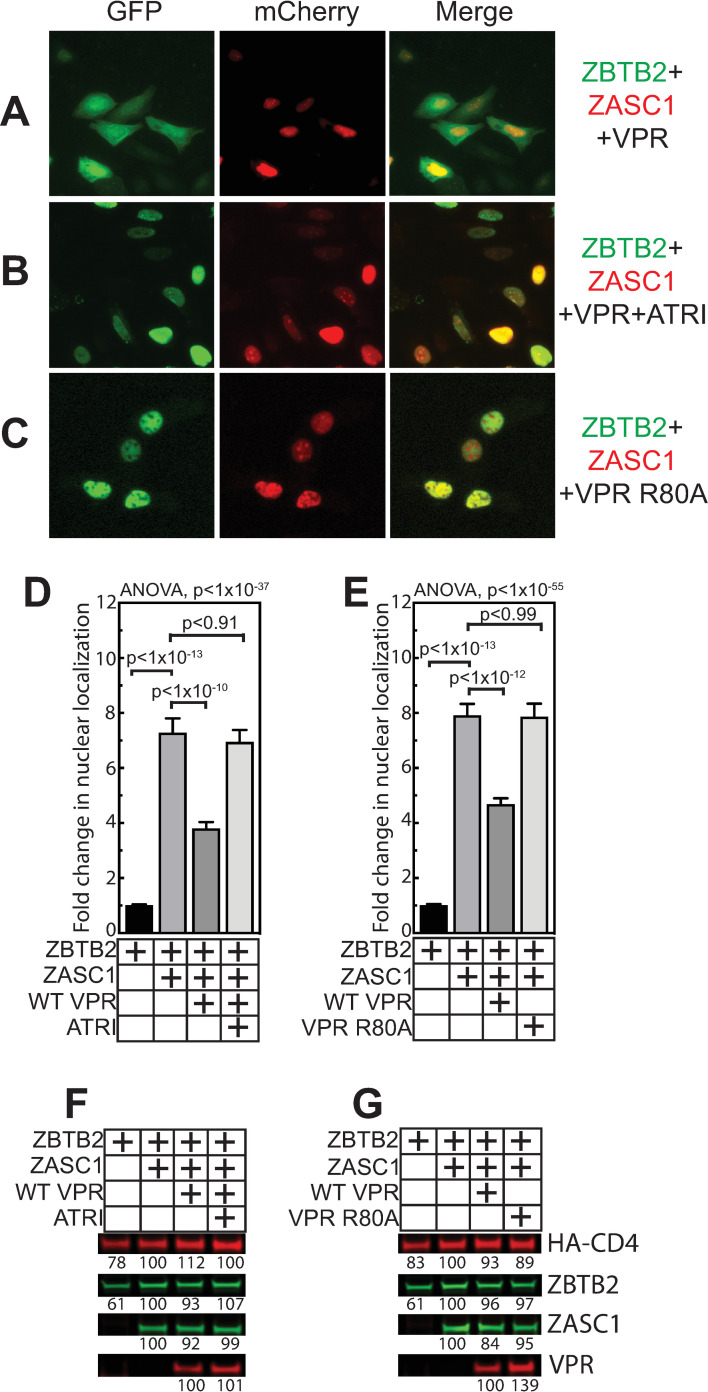
Vpr activation of the ATR kinase pathway regulates ZBTB2 localization. U2OS cells were transfected with GFP-ZBTB2 and mCherry-ZASC1 and imaged 24 hours post transfection. Co-expression of WT Vpr (A) resulted in a primarily cytoplasmic ZBTB2 localization which was reversed (B) by treating with 10 μM of ATR inhibitor (CAS 905973-89-9). (C) Co-expression of the cell-cycle arrest-deficient Vpr R80A mutant failed to relocalize ZBTB2 to the nucleus. (D and E) To quantify the effects observed in (A-C), independent transfections were performed with the indicated conditions and the relative ZBTB2 fluorescence signals in the nucleus and a nucleus-adjacent cytoplasmic ring were measured in ≥100 cells and the fold change in nuclear to cytoplasmic signal reported. See Fig 9A and 9B for representative results of cells expressing ZBTB2 alone or in combination with ZASC1. (D) presents the effects of WT Vpr expression and the effect of 10 μM ATR inhibitor, and (E) presents the effects expressing the Vpr R80A cell cycle arrest deficient mutant. Error bars indicate the standard deviation of the data in all panels. ANOVA analysis was performed and for P-values < 0.05 a Tukey’s HSD was performed and relevant P-values reported. Western blots showing the effects of (F) Vpr and ATR inhibitor or (G) WT and R80A Vpr on the levels of ZASC1, ZBTB2 and a HA-tagged CD4 control protein. Numbers indicate band intensity relative to lane expressing both ZASC1 and ZBTB2. Results are representative of three independent experiments.

Similarly, co-transfecting GFP-ZBTB2, mCherry-ZASC1 and Vpr resulted in relocalizing ZBTB2 from the nucleus to the cytoplasm (Fig 11A and 11D), which could be blocked with ATR kinase inhibitor treatment (Fig 11B and 11D). Importantly, the Vpr R80A mutant deficient in cell-cycle arrest did not cause relocalization of ZBTB2 (Fig 11C and 11E). Vpr expression was unaltered by the ATRi inhibitor (Fig 11F) and both the WT and R80A Vpr variants expressed to similar levels (Fig 11G). These data imply that activating the ATR pathway by DNA damaging agents or Vpr expression alter ZASC1:ZBTB2 interaction.

#### ATR activation by Vpr or DNA damaging agents inhibit ZBTB2 recruitment and function at the HIV-1 promoter

**Fig 12:**
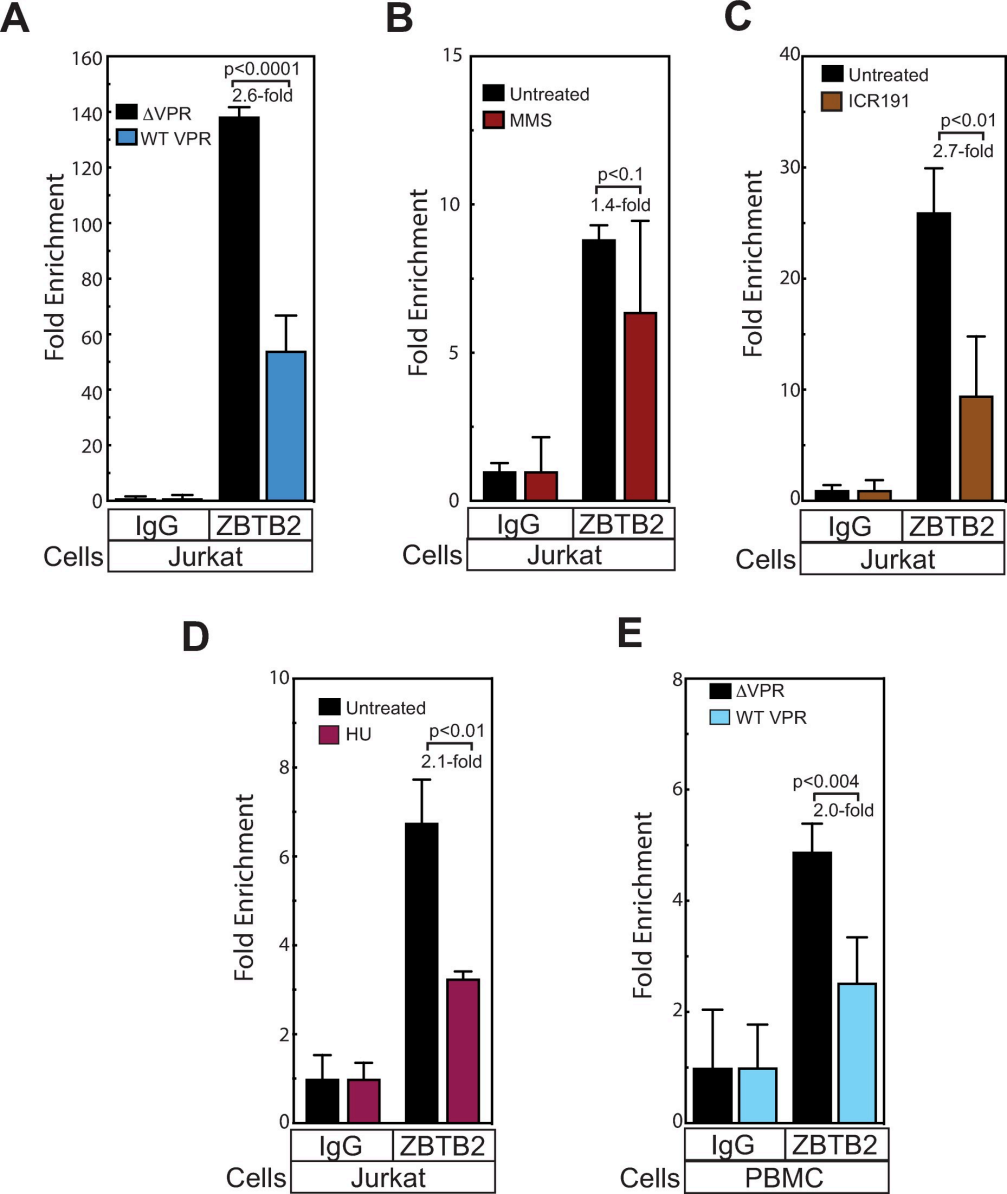
Activation of the Vpr/ATR pathway impairs ZBTB2 recruitment to the HIV-1 promoter. (A) ChIP analysis of ZBTB2 recruitment to the HIV-1 promoter in Jurkat cells infected with an HIV-1 NL43 variant that either expresses Vpr or lack Vpr. (B) ChIP analysis of ZBTB2 recruitment to the HIV-1 promoter in Jurkat cells treated with 8 μM methyl methanesulfonate (MMS) (C) 25 mM Hydroxyurea or (D) 4μg/ml ICR-191 and infected with an HIV-1 vector. (E) ChIP analysis of ZBTB2 recruitment to the HIV-1 promoter in primary PBMCs stimulated with CD3/CD28 beads and infected with an NL43 variants either expresses or lacks Vpr. Immunoprecipitation and Real-time PCR analysis were performed and normalized to input controls and reported as percent of starting material for immunoprecipitation. Fold enrichment of experimental IPs relative to IgG controls is reported below the graph. The data shown are the average mean values obtained in an experiment performed with quadruplicate samples and are representative of three independent experiments. Error bars indicate the standard deviation of the data in all panels. P-values were calculated using a standard Student’s t-test and significant changes relative to relevant bracketed comparisons indicated.

**Fig 13:**
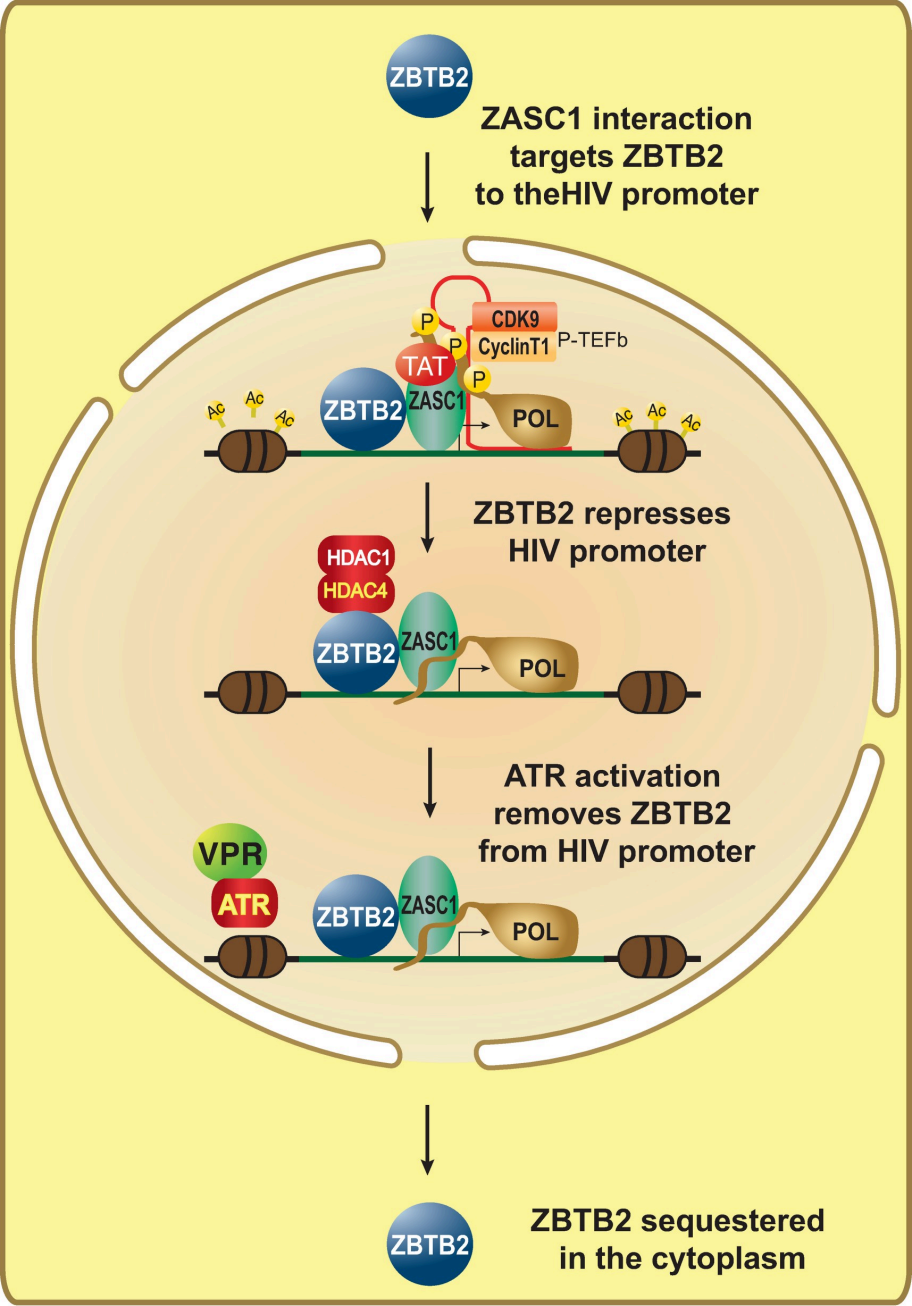
Model for ZASC1 and ZBTB2 regulation of HIV-1 transcription. In the presence of Vpr or other activation of the DNA damage response, ZBTB2 is exported from the nucleus, ZASC1 binds the HIV-1 LTR promoter and facilitates the assembly of the TAT P-TEFb elongation complex. Under conditions of low Vpr, ZBTB2 is recruited to the HIV-1 promoter by ZASC1, binds cellular HDAC complexes and represses HIV-1 transcription.

To determine if Vpr and/or ATR activities can disrupt ZBTB2 function as well as localization, we performed ChIP analysis on cells infected with HIV vectors containing either a WT *vpr* gene or a *vpr* gene with a nonsense mutation (ΔVpr). As predicted, Vpr expression reduced ZBTB2 recruitment to the LTR promoter by a statistically significant 2.6-fold (Fig 12A). However, in cells challenged with a ΔVpr vector (Fig 12B), the DNA damaging agent methyl methanesulfonate (MMS) reduced ZBTB2 recruitment by only 1.4-fold, which was not statistically significant, due in part to increased variability observed with both the control IgG and anti-ZBTB2 immunoprecipitations and possibly associated with toxicity. To further explore the prior connection with DNA damage responses, we also tested DDR-inducing agents [64,65] ICR191 (Fig 12C) and hydroxyurea (Fig 12D), which paralleled Vpr in inhibiting ZBTB2 recruitment to the LTR promoter by a statistically significant 2.7- and 2.1-fold, respectively. While we did not observe any obvious cell loss with ICR191 or hydroxyurea, we cannot completely rule out that some cytotoxicity of these compounds might have contributed to the results. Nevertheless, activation of the DDR by these chemicals or by Vpr consistently reduce the amount of ZBTB2 detected at the promoter. To determine if the inhibitory effect of Vpr on ZBTB2 recruitment was observed in primary T cells, we induced T cell proliferation by treating PBMCs with CD3/CD28 beads [66] and then challenging with either a WT or ΔVpr HIV-1 vector (Fig 12E). The ΔVpr HIV-1 vector showed a 4.8-fold enrichment in ZBTB2 recruitment to the HIV-1 promoter in primary cells, while expressing Vpr induced statistically significant reduction of this enrichment to 2.2-fold. Thus, we have shown that ZASC1 interacts with ZBTB2, which in turn recruits cellular HDACs to the HIV-1 promoter, leading to histone deacetylation and repression of HIV-1 gene expression (Fig 13 top). This inhibition of gene expression can be relieved by activating the ATR kinase pathway by HIV-1 Vpr or DNA damage (Fig 13 bottom).

## Discussion

### ZBTB2 is a novel repressor of the HIV-1 promoter

Here we have shown by multiple lines of evidence that ZBTB2 is a novel, ZASC1-recruited repressor of gene expression from integrated HIV-1 proviral DNA. In addition, ZBTB2 expression repressed the plasmid-borne HIV-1 LTR promoter in transient transfection assays (Fig 3A). siRNA knockdown and CRISPR/Cas9 knockout of ZBTB2 each resulted in dramatic increases in HIV-1 expression (Figs 3B and 5) in the absence of Vpr expression. Since the same HIV-1 repression phenotype was reproduced with mechanistically distinct approaches, it is highly unlikely that this phenotype is due to an off-target effect. Further, ZASC1 activation (Fig 6A) and ZBTB2 repression (Fig 6B) were restored by exogenous protein expression in their respective CRISPR/Cas9 knockout lines. Moreover, we showed that repression of the HIV-1 promoter was dependent on the ZBTB2 POZ domain (Fig 3A), which is required for interaction with cellular HDACs (Fig 7). This is consistent with previous work that the ZBTB2 POZ domain recruits cellular co-repressors [51]. Interestingly, this previous work showed an interaction with HDAC3 but did not test other HDACs. Our results showed weak interaction between ZBTB2 and HDAC3, but much stronger interaction of ZBTB2 with HDAC1, 4, 7 and 9 (Fig 7A and 7B). In further confirmation of this ZBTB2 interaction with cellular HDACs, both ZBTB2 and ZASC1 co-purified with HDAC1 by IP-mass spectrometry [48]. Interestingly, HDAC4 interacts with class I HDAC-containing repressor complexes [67–69], suggesting that the ZBTB2 POZ domain may actually serve to organize a larger co-repressor complex containing multiple HDACs on the HIV-1 promoter. Functionally, loss of ZBTB2 recruitment to the HIV promoter resulted in increased histone acetylation (Fig 9A and 9B) and a moderate loss of total histone H3 (Fig 9C), correlating with an increase in HIV-1 gene expression and replication (Figs 3B and 5). This is consistent with the known effects of nucleosome remodeling after histone acetylation, including the loss of a nucleosome specifically positioned overlapping the transcripton start stie [61–63]. Thus, ZBTB2 localization to the HIV-1 promoter results in cellular HDAC recruitment, histone deacetylation, chromatin condensation and repression of viral gene expression.

### ZBTB2 is recruited and regulated by ZASC1

Previously we showed that ZASC1 is a sequence-specific DNA binding protein that stimulates transcription elongation from the HIV-1 LTR by TAR-independent recruitment of TAT and P-TEFb to the LTR promoter DNA [38]. Here we demonstrated that ZASC1 and ZBTB2 interact through zinc finger 1 of ZBTB2 and Zinc finger 6 of ZASC1 (Fig 1). In addition, we observed that co-expressing ZASC1 and ZBTB2 results in relocalizing ZBTB2 from the cytoplasm to the nucleus, and this relocalization is dependent on the ZBTB2:ZASC1 interaction domains (Fig 2). Importantly, we showed that ZASC1 is required for high-level recruitment of ZBTB2 to the HIV-1 promoter (Fig 8B and 8C). Thus, ZASC1, an enhancer of transcription elongation, under some conditions specifically recruits a transcriptional repressor to the HIV-1 promoter.

ZBTB2 recruitment to promoters in the DNA damage pathway has been linked to ZBTB2 binding to GC-rich DNA, in particular SP1 sites [51]. Additionally, it was reported that ZBTB2 binds to SP1 protein [51]. Interestingly, the HIV-1 promoter has three highly conserved SP1 sites [2,59]. In our hands, under in vivo conditions where ZASC1 and ZBTB2 interact strongly, ZBTB2 interaction with SP1 is dramatically weaker (S3A Fig). In addition, we see no relocalization of ZBTB2 upon co-expressing SP1 (Fig 2L). However, even though our data imply that ZASC1 is the primary determinant of ZBTB2 recruitment for HIV, it is possible that interactions with SP1, other unknown cellular proteins, or ZBTB2’s ability to bind to GC-rich DNA [51] may also contribute to ZBTB2 recruitment to the HIV promoter or cellular promoters.

Recruitment of ZBTB2:HDAC complexes by ZASC1 could explain why ZASC1 has been reported to be a transcriptional repressor in some systems [44]. Further, both HDAC4 [70] and ZASC1 [43] have been linked to inherited ataxias. Based on the findings reported here, it is possible that dysregulation of ZASC1:ZBTB2:HDAC4 interactions contributes to these ataxias. This possibility is enhanced by strong links of ataxias to mutations in DNA damage response pathways, including ATR and ATM [71,72] and our observations that the DNA damage response regulates ZASC1:ZBTB2 interaction, as noted below.

### ZBTB2 function is regulated by Vpr/ATR/DDR

HIV-1 virion protein Vpr induces cell cycle arrest by activating the ATR pathway [19,20] and initiating the DNA damage response (DDR). The ability of Vpr to stimulate HIV-1 gene expression is primarily due to the increase in HIV-1 LTR promoter activity in G2-arrested cells [6]. Our studies revealed multiple lines of evidence supporting integrated roles for Vpr, ATR and the DDR in regulating ZASC1 and ZBTB2 function. First, Jurkat and SupT1 cells with ZBTB2 deleted showed markedly increased expression (4.8- and 11-fold, respectively) of the HIV-1 promoter relative to WT cells when challenged with vectors lacking Vpr, but not when challenged with virions containing Vpr (Fig 5A). Similarly, siRNA depletion of ZBTB2 enhanced HIV-1 promoter activity by 4-fold only in the absence of Vpr (Fig 3B). Consistent with these indications of linkage between ZBTB2 and Vpr functions in HIV-1 transcription, in transient reporter assays, exogenous Vpr expression had no effect on HIV-1 promoter activity in either the ΔZASC1 or ΔZBTB2 cell lines (Fig 6C and 6D). These losses of Vpr responsiveness imply that a significant portion of Vpr’s effect on HIV-1 transcription is mediated through effects on ZBTB2, which as noted above is dependent on ZASC1. Moreover, we showed that ZASC1 or ZBTB2 knockout did not significantly alter 2LTR circle formation or, for a ΔVpr virus, levels of integrated proviral cDNA (S2A and S2B Fig, respectively), ruling out pre-integration and integration effects as a cause for altered transcription.

In line with these effects, inducing the DDR through ATR activation by Vpr or DNA damaging agents interfered with the ability of ZASC1 to relocalize ZBTB2 to the nucleus (Figs 10 and 11). This effect could be reversed with chemical inhibitors of ATR (Figs 10 and 11). In parallel, ATR activation by Vpr or DNA damaging agents inhibited ZASC1 recruitment of ZBTB2 to the HIV-1 promoter (Fig 12). Interestingly, we found no interaction between Vpr and ZBTB2 by co-immunoprecipitation (S3B Fig), suggesting that the effects of Vpr are mediated indirectly through Vpr activation of ATR. Further, ZBTB2 relocalization was a relatively rapid event visible 4 hours post DNA damaging agent treatment, suggesting that ATR activation is sufficient for loss of ZBTB2 repression without full cell cycle arrest. Together, these data support a model in which Vpr/ATR regulates a switch between two transcriptional states of the HIV-1 LTR promoter (Fig 13). In the absence of ATR activation, ZASC1 binds ZBTB2, cellular HDACs are recruited to the promoter and histones are deacetylated and condensed, repressing transcription. Conversely, when Vpr or DNA damage activates ATR, ZBTB2 or a ZBTB2 cofactor is phosphorylated to initiate removal of the ZBTB2:HDAC complex from the promoter, increasing basal transcription and facilitating ZASC1 recruitment of TAT and p-TEFb to nascent TAR RNA to stimulate transcription elongation. The significant but partial block to ZBTB2 recruitment observed with Vpr and DNA damaging agents (Fig 12) and residual promoter-associated ZBTB2 in ΔZASC1 cells (Fig 8C) suggest that ATR activation may initiate a multistep process of ZBTB2 inactivation and removal that includes loss of interaction with ZASC1, subsequent release from other chromatin-associated ZBTB2 interaction partners such as HDAC complexes and SP1, and loss of DNA association. Once fully free from the promoter, ZBTB2 can exit the nucleus and be sequestered in cytoplasm. Vpr and the DDR thus may initiate targeted removal of ZBTB2 from the LTR promoter, but other cellular processes and steps are likely necessary for full ZBTB2 release and sequestration in the cytoplasm.

### ZBTB2/ZASC1 integrate TAT and Vpr activities

The interaction between ZASC1 and ZBTB2 forms a regulatory nexus between HIV-1 TAT and Vpr (Fig 13). Vpr and TAT have long been known to synergistically act to maximize HIV-1 gene expression [6–8]. Incoming virions contain significant amounts of Vpr that are sufficient to induce cell cycle arrest [73] and stimulate HIV-1 gene expression (Figs 3B, 5, 6C and 6D). This could be especially critical under conditions of low TAT, such as just after proviral establishment. Vpr activation of ATR removes repressive ZBTB2 from the HIV-1 promoter, prevents HDAC-induced chromatin condensation and makes ZASC1-mediated recruitment of TAT and P-TEFb more effective. Conversely, insufficient ATR activation by Vpr or a cellular environment not conducive to active replication, such as transitioning to a memory T cell, results in ZBTB2 recruitment, histone deacetylation, chromatin condensation, repression of gene expression and ultimately latency. These data also suggest that regulation of ZBTB2/ZASC1 interaction may play an important role in HIV-1 reactivation from latency, particularly since DNA damage and Vpr can efficiently reactivate latent HIV-1 [15,16,27,28].

Both ZBTB2 and ZASC1 have strong links to cancer [39–42,53–55]. In addition to our findings that ZBTB2 is regulated by the DNA damage pathway (Figs 3B, 5, 6C, 6D, 10, 11 and 12), ZBTB2 is implicated in regulating genes in this pathway [51]. ZBTB2 also was recently identified as a potential oncogene in a screen of colorectal cancers with microsatellite instability [53]. Mutational hotspots were observed within ZBTB2 zinc finger 1 that is required for ZASC1 binding, suggesting that dysregulation of ZASC1/ZBTB2 interaction might contribute to malignancy. IP-mass spec experiments with tagged ZBTB2 identified numerous putative interacting proteins [53], including ZASC1 and Vpr interacting protein VprBP/DCAF1. Interestingly, in our experiments we have never observed expression of Vpr, either in transient transfection or infections, reproducibly affecting the levels of either ZASC1 or ZBTB2 (see Fig 11F and 11G), suggesting that Vpr’s modulation of the proteosome is not regulating ZASC1 or ZBTB2. While minor (≤10%) fluctuations in ZBTB2 and ZASC1 levels can occasionally be seen in western blotting in the presence of Vpr, this could be due to differences in the stability of cytoplasmic vs nuclear ZBTB2. These effects are also minor compared to Vpr effects on ZBTB2 localization and the combinatorial effects of Vpr and ZBTB2 on HIV-1 transcription. These results support our findings and suggest that ZASC1 and ZBTB2 have important effects on the DDR and potentially oncogenesis. Since Vpr degrades chromatin-associated class I HDACs [15,16], a combination of direct HDAC degradation plus loss of ZBTB2 recruitment of HDACs could cooperatively enhance Vpr stimulation of HIV-1 transcription. Future work will include elucidating cellular promoters regulated by ZASC1 and ZBTB2, and the roles of ZASC1 and ZBTB2 in the cellular DDR as well as in HIV-1 latency and reactivation. Reactivation is a potentially attractive approach for eliminating the long-lived latent pool of HiV-1 infected cells [74]. Understanding the specific interactions between HIV-1 and cellular factors during replication and reactivation will improve such approaches by targeting them more specifically to the virus and reducing side effects.

## Materials and methods

### Plasmids and viral vectors

The viral genome pNL4-3.Luc.R-E- and ZASC1 binding site variants have been previously described [75,76] and is the base vector for all HIV-1 genomes used in this study. Briefly, this is the NL43 genome with nonsense mutations inserted in the envelope and *vpr* genes and firefly luciferase (fLuc) inserted in the Nef locus. pNL4-3.Luc.R-E- variants with mCherry instead of fLuc in the Nef locus with either WT or mutant *vpr* genes were a kind gift from Nate Sherer [77]. To make pNL4-3.Luc.R-E- variants (HIV-Luc, HIV-Luc ΔVpr, and HIV-Luc, ΔVpr mZBS) with either WT or mutant *vpr* genes, and WT or mZBS LTR promoters, that encode GFP-nanoLuciferase (NLuc) fusion protein in the Nef locus, a GFP-NLuc geneblock (IDT, Coralville, IA) was cloned into the Nef locus of the above pNL4-3.Luc.R-E- derivatives as a *NotI/XhoI* cassette. ZASC1 and ZBTB2 expression vectors were generated by PCR amplification of coding sequence (ZASC1(ZNF639):IMAGE#4794621, ZBTB2: IMAGE#4577244) from commercially available cDNAs (Open Biosystems, Huntsville, AL) and cloning into pCMV-TNT (Promega, Madison, WI). Epitope tags were added by traditional subcloning. Replication competent NL4-3 variants were kind gifts from Nate Sherer.

Melanie Ott kindly provided the plasmids pEV280, which expresses WT two-exon TAT with a C-terminal FLAG-tag from the HCMV promoter [78] through Addgene Incorporated.

Flag-tagged versions of mammalian HDAC1, 3,4,5,6,7,8 were a kind gift from Eric Verdin through Addgene (plasmids 13820, 13819, 13821, 13822, 13823, 13824, 13825, respectively).

FLAG-tagged HDAC2, and 9 were PCR amplified from a cDNA clone from DNASU (plasmids HsCD00005288, HsCD00351633 and cloned into a mammalian expression vector in frame with a FLAG epitope. The HDAC10-V5 tagged expression vector was obtained from DNASU (DNASU HsCD00443571). Plasmids with retroviral promoters for reporter gene analysis were previously described [45] and contain the HIV-1 U3 and TAR element from pNL4-3.Luc.R-E- cloned into pGluc-Basic (NEB, Ipswich, MA). The plasmid expressing *cypridinia* luciferase under the control of the HSV-1 tymidine kinase promoter (ptk-cLuc) was purchased commercially (NEB, Ipswich, MA). pEGFP-N1 expressing GFP under the control of the CMV promoter was purchased commercially (Clontech, Mountain View, CA). The Vpr expression plasmid pCMV-Vpr was made by ordering a geneblock with the either the WT NL43 Vpr coding sequence or the R80A mutation (IDT, Coralville, IA) and cloning it into the pCMV-TNT vector (Promega, Madison, WI). All clones were validated by sequencing.

### Cell culture and virus production

Human embryonic Kidney 293T cells (HEK293), U2OS, and Jurkat and were purchased from ATCC (Manassas, VA) while SUPT1 cells were obtained from the NIH AIDS Reagent program. All cell lines were maintained at low passage number as previously described [75]. The procedures used to produce the retroviral vectors and titer each viral stock are described in detail elsewhere [75].

Primary T-cells and PBMCs were obtained from Sanguine BioSciences (Santa Monica, CA) or ZEN-Bio (Research Triangle Park, NC), thawed into RPMI 1640 supplemented with 10% FBS and penicillin/streptomycin. Cells were stimulated with a 1:1 ratio of anti-CD3 and anti-CD28 beads (Invitrogen, Carlsbad, CA) following manufacturer’s instructions and expanded in the presence of 20U/ml of IL-2 (Sigma, Saint Louis, MO). Cells were infected by spinoculation at 3000 xg for 2 hrs in multi-well plates at an MOI of 3 or by infection in the presence of 6μg/ml DEAE dextran. Experiments with primary T-cells were repeated with cells from at least two different donors.

### Assays of viral infection

Quantitative chemiluminescent infection assays were performed as previously described [75,79]. Briefly, 96 well plates were seeded at 1X10^4^ cells/well for each cell line tested. The cells were incubated with an approximate MOI of 1 transducing unit, in the presence of 6μg/ml DEAE dextran for 48 hpi. Cells were washed four times with PBS and four wells were assayed for firefly luciferase (fLuc) activity using the Britelite reagent (PerkinElmer, Boston, MA) or NanoLuciferase (NLuc) (Promega, Madison, WI) according to the manufacturer’s instructions. The other four wells were assayed for cell number and cell viability using CellTiter-Glo reagent (Promega, Madison, WI). The results obtained were normalized for relative cell number. Replication of WT HIV-1 or HIV-1 with a Vpr nonsense mutation (ΔVpr) in WT or ΔZASC1 Jurkat cells. Viral titers were calculated by determining the p24 levels released into the supernatant using the Perkin Elmer Alliance HIV-1 p24 ELISA kit (NEK050001KT) following manufactures instruction and correlated to infectious units by X-gal staining on TZMBL reporter cells using the Sigma β-galactosidase reporter gene staining kit (GALS-1KT) following manufactures instructions. For replication assays, cells (1X10^6^) were infected with the indicated virus at an moi of 0.005. Supernatant was titered on TZMBL reporter cells at the indicated time points and infectious units counted by X-gal staining.

### Transient transfection assays

For transient promoter activation assays effector and reporter plasmids were transfected into Jurkat cells using 10 μl tips and the Neon electroporation system (Invitrogen, Carlsbad, CA) following manufacturer’s instructions. Briefly, 2.0 μg of plasmid was mixed with 5X10^5^ Jurkat cells in 10 μl buffer R. Cells were electroporated in Buffer E with three pulses at 1600 Volts and 10 ms pulse width. Cells were allowed to recover in 0.5 ml RPMI supplemented with 10% FBS without antibiotics for 24 hrs in a 24-well plate. Cells were counted and then equivalent number of cells were transferred to quadruplicate wells of a 96-well plate, incubated for an additional 24 hours and assayed for reporter gene expression. For transient promoter activation assays, 100 ng of the retroviral reporter construct, 250 ng of a GFP expression plasmid, 350 ng of a *cypridinia* luciferase expression plasmid were included in all transfections. ZBTB2 expression plasmids and TAT expression plasmids were included at 1000 and 100 ng/well, respectively. Vector plasmid DNA or Calf thymus DNA was used to maintain a constant 2000 ng/well in each well. Two days post-transfection, 10 μl of media was removed, diluted with 40 μl of PBS and assayed for secreted gaussia luciferase (gluc) by injecting 30 μl coelenterazine solution (*Renilla* luciferase assay system, Promega, Madison, WI), waiting 1.6s and then reading luminescence for 1s. *Cypridinia* luciferase (cLuc) activity from the internal control plasmid was determined using the BioLux *cypridinia* luciferase kit (NEB, Ipswich, MA) according to the manufacturer’s instructions. The activity of the retroviral promoter in each well was then expressed as the relative light units of gLuc or the ratio of gLuc:cLuc.

### siRNA knockdown

Control and siZBTB2 targeting siRNAs were transfected into SUPT1 cells using 100 μl tips and the Neon electroporation system (Invitrogen, Carlsbad, CA) following manufacturer’s instructions. Briefly, 600 nM of siRNA was mixed with 5X10^6^ Jurkat or SUPT1 cells in 100 μl buffer R. Cells were electroporated in Buffer E2 with one pulse at 1625 Volts and 20ms pulse width. Cells were allowed to recover in RPMI supplemented with 10% FBS without antibiotics for 48 hrs, counted and then 1 X10^4^ cells were transferred to a 96 well plate and challenged with VSV-G pseudotyped NL43E-R-Luc and assayed for infectivity as described above. For complementation with virion delivered Vpr, VSV-G pseudotyped NL43E-R- was prepared as normal but 2 μg of Vpr expression plasmid was co-transfected into the producer cells. The average infection of four control knockdowns with siRNAs targeting MALAT, GFP, and two targeting CD4 (Invitrogen siRNAs 4455877, AM4626, 4392420, 4392420, respectively) were compared to the siRNA knockdown of ZBTB2 (Silencer select 4427037, Invitrogen, Carlsbad, CA). Infection of control siRNA transfections varied by less than 20%. Western blot knockdown comparison was of siRNA knockdown of siGFP relative to siZBTB2.

### CRISPR/Cas9 HDR directed knockout

CRISPR/Cas9 plasmids targeting ZASC1 (sc-41729) and ZBTB2 (sc-412860) and corresponding homologous directed repair plasmids (sc-41729-HDR, sc-412860-HDR) were obtained commercially (Santa Cruz Biotechnology, Santa Cruz, CA). Equivalent amounts of CRISPR/Cas9 and HDR plasmids were transfected using 100 μl tips and the Neon electroporation system (Invitrogen, Carlsbad, CA) following manufacturer’s instructions. Briefly, 10 μg of plasmid was mixed with 5X10^6^ Jurkat or SUPT1 cells in 100 μl buffer R. Cells were electroporated in Buffer E2 with one pulse at 1775 Volts and 20ms pulse width. Cells were allowed to recover in RPMI supplemented with 10% FBS without antibiotics for 48 hrs. Cells where homologous recombination had knocked out the targeted gene and inserted the puromycin resistance cassette were selected for in RPMI supplemented with 2 μg/ml Puromycin for two weeks. Loss of expression was determined by western blotting.

### Immunoprecipitation and Western blot analysis

Cells (1 X10^7^) were lysed in 500 μl ice cold Tris buffered saline NP40 (TBSN) [150 mM NaCl, 50 mM TRIS (pH 7.5), 1 mM EDTA, 2 mM β-mercaptoethanol, 0.5% NP40, 1X HALT protease inhibitor cocktail (Invitrogen, Carlsbad, CA)] buffer. Cells were allowed to lyse on ice for 10 min, and nuclei were pelleted at 20,000XG for 10 min, supernatant was transferred to a new tube and 50 μl (10%) of the input material (Input) was removed for SDS-PAGE. The remaining sample was immunoprecipitated with EZview Red Anti-HA, anti-FLAG affinity gels (Sigma, Saint Louis, MO), or anti-Myc agarose (Santa Cruz Biotechnology, Santa Cruz, CA) in the presence of 2,000 gel units micrococcal nuclease (NEB, Ipswich, MA) for 1 hour. The samples were washed three times in TBSN, and the beads resuspended in 50 μl SDS-PAGE loading buffer. The samples were boiled, equivalent volumes of Input and IP elution were separated by SDS-PAGE, transferred to PVDF membrane and blotted with antibodies raised against either the HA epitope (Bethyl laboratories, A190-106A or A190-108A, Montgomery, TX), the FLAG epitope (Sigma, F7425, Saint Louis, MO), the Myc epitope (Bethyl laboratories, A190-103A or A190-105A, Montgomery, TX), human ZASC1 (Bethyl Laboratories, A302-400A, Montgomery, TX or Abcam, AB185106, Cambridge, MA), human ZBTB2 (Novus Biologicals, NBP1-88787, Centennial, CO) human Lamin-B (Santa Cruz Biotechnology, sc-377000, Santa Cruz, CA) or GFP (Santa Cruz Biotechnology, sc-9996, Santa Cruz, CA). The blots were washed, treated with the appropriate secondary far-red antibody (LI-COR Biosciences, Lincon, NE), and fluorescent signal was detected and quantified on an Odyssey CLx (LI-COR Biosciences, Lincoln, NE) using ImageStudio software. In general, ZASC1 and ZBTB2 WT immunoprecipitations recovered between 50 to 70% of the antibody targeted protein and co-immunoprecipitated between and 10 to 30% of interacting protein. For endogenous protein immunoprecipitation, cells (1X10^7^) were harvested and immunoprecipitated as described above except they were incubated with incubated with 2 μg anti-ZASC1 (Bethyl A302-401A) or ZBTB2 (Bethyl A303-261A) for 1 hour and Pierce magnetic Protein A/G (Cat#88803) overnight. Samples were processed as above and western blotting was done using anti-ZASC1 (Bethyl A302-400A) or ZBTB2 (Bethyl A303-262A) and Protein A DyLight 800 (Rockland, Cat# PA00-45). Virus release into media was detected using the monoclonal anti-p24 capsid antibody derived from the HIV-1 p24 Hybridoma (183-H12-5C) from Dr. Bruce Chesebro [80] obtained through the AIDS Research and Reference Reagent Program, Division of AIDS, NIAID, NIH.

### Chromatin immunoprecipitation (ChIP) Real-time PCR

ChIP conditions were performed using a previously described [81] protocol with the following modifications. Formaldehyde crosslinked (0.5%, 5 min.) cells (2X10^7^) were sonicated in cell lysis wash buffer (CLB) for 70 cycles of 30s on 45s off in a Misonix Q700 cup horn Sonicator (Qsonica, Newtown, CT) at 95% power. To determine the effect of DNA damaging agents, 48 hours post-infection cells were treated with 8 mM methyl methanesulfonate (MMS) and chromatin was harvested 16 hrs post treatment. Sonicated samples were incubated overnight with non-specific rabbit IgG (Millipore, Billerica, MA), rabbit anti-ZASC antibody A302-401A (Bethyl laboratories, Montgomery, TX), anti-ZBTB2 antibody A303-261A-1 (Bethyl laboratories, Montgomery, TX) rabbit anti-SP1 A300-133A (Bethyl laboratories), rabbit anti-acetylated Histone H3 ab47915 (Abcam, Cambridge, MA), rabbit anti-pan Histone H3 05–928 (Millipore, Burlington, MA). Immunocomplexes were purified with Protein A/G magnetic beads (Pierce, Rockford, IL). Immunoprecipitated DNA was analyzed by quantitative real-time PCR on a CFX96 (Bio-Rad, Hercules, CA) using probes with a 5’ 6-FAM dye and a 3’ Iowa black and internal ZEN Quenchers (IDT, Coralville, IA) in SsoFast Universal Probes Supermix with low ROX following manufacturer’s recommendations (Bio-Rad, Hercules, CA). Standard curves were generated using plasmids containing the appropriate amplicon (full length NL4-3 provirus for HIV-1 sequences). The primers and probes used for analysis are: HIV start Fwd: GGGAGTGGCGAGCCCTCAG HIV start rev: CAGGCTCAGATCTGGTCTAAC, HIV probe: CTTTTTGCCTGTACTGGGTCTCTCT, Vif Fwd: ATGGCAGGTGATGATTGTGTG, Vif Rev: GCTTTCCTTGAAATATACATATG, Vif Probe: CCATGTGTTAATCCTCATCCTGTC, Gene Desert Fwd: GGCGACTTGACTTCAGAGACAATG, Gene Desert Rev: GGAAAGAGGATGAGAAAGGCAGG, Gene Desert Probe: GAGGCGCGACTTGACTTCAA.

### 2LTR Circle Assay and integrated provirus analysis

Cells (1X10^6^) were infected at an MOI of 1 with replication competent HIV-1, at 48 hours post infection, total DNA was harvested using the DNeasy kit from Qiagen. Quantitative real-time PCR was performed as described [58] using the following primers: HIV-1 2LTR Fwd primer: AACTAGGGAACCCACTGCTTAAG, HIV-1 2LTR Rev primer: TCCACAGATCAAGGATATCTTGTC, HIV-1 2LTR probe: ACACTACTTTGAGCACTCAAGGCAAGCTTT, Cell number PBDG1 Fwd: AAGGGATTCACTCAGGCTCTTTC, PBDG1 Rev: GGCATGTTCAAGCTCCTTGG, PBDG1 probe: CCGGCAGATTGGAGAGAAAAGCCTGT

Integration of VPR deletion virus was assayed by challenging cells (1X10^6^) at an MOI of 5 (based on GFP levels in HEK293 cells) of VSV-G pseudotyped HIV-1 vector with nonsense mutations in envelope and Vpr, GFP-Nanoluc in the NEF locus and passaging for 7 days to allow for loss of unintegrated DNA. Total cellular DNA was isolated from 1X10^6^ cells and HIV DNA was quantitated by real-time Quantitative PCR with the HIV promoter primer (HIV start Fwd, HIV probe, HIV start Rev) set used as described above for the ChiP experiments.

### Fluorescent microscopy

U2OS cells (4X10^4^ cells/well) were transfected in a 12-well chamber slide (Ibidi, Fichburg, WI) with 150 ng total DNA and 0.45 μl Transit LT1 (Mirus, Madison, WI) following manufacturer’s instructions. Cells were transfected with 75 ng each of an expression plasmid encoding GFP-ZASC1 and either a nuclear mCherry or an mCherry-ZASC1 fusion protein. For UV treatment, 20 hours post-transfection, media was replaced with PBS, and the slides were placed on a UV transilluminator and exposed to 320 nm UV light for 5 minutes. PBS was replaced with media and cells were imaged 4 hours post-UV treatment. ATR inhibitor (CAS 905973-89-9, (Santa Cruz Biotechnology, Santa Cruz, CA) was added to PBS and to media at a final concentration of 10 μM just prior to UV irradiation and maintained in media after treatment. Cells were imaged with a 40X objective (Fig 2) or 10X objective (Figs 10 and 11) on a Biotek Cytation 5 automated microscope (BioTek, Winooski, VT) 24 hrs post transfection. For quantitation, cells were transfected with GFP-ZBTB2, ZASC1 and Vpr expression plasmids, 20 hours post transfection, cells were treated with UV light or chemicals, fixed and stained with DAPI. A 3X3 montage of 10X fields were collected on a Biotek Cytation 5 automated microscope (BioTek, Winooski, VT). Single cell analysis by segmenting the nuclear and cytoplasmic compartments was performed using a custom KNIME image processing workflow. The KNIME workflow accepts multi-channel images, where a nuclear marker (*e*.*g*. DAPI stain) is in channel 1 followed by *n* channels, and outputs per cell measurements of both the nuclear and cytoplasmic compartments across multiple channels. Briefly, the cells are identified by first illumination correcting the nuclear channel followed by thresholding the signal. Next, the nuclear threshold is smoothed out by the Fill Holes node before finally being processed by the Waehlby Cell Clump Splitter node to separate closely clumped nuclei. The nuclear masks generated are also used to generate a dilated cytoplasmic ring around the nucleus. Together the nuclear masks and the resulting nucleus-adjacent cytoplasmic reference ring along with the additional channels were processed to obtain the mean fluorescence intensity (MFI) of the nucleus and cytoplasm. All results are written as a.CSV file which was then further processed in Microsoft Excel.

### Statistical analysis

For comparisons with three or more groups, a one-way ANOVA analysis was performed using Microsoft Excel. If this identified that a statistically significant difference existed within the group (p<0.05), a further post hoc test was done using Tukey’s Honestly Significant Difference test (HSD) [82,83] in Excel. If only two groups were compared, Student’s T-test in Excel was used. ANOVA results and relevant Tukey’s HSD and T-test comparisons are shown on figures, unless p>0.05 for the ANOVA, in which case only the ANOVA results are shown.

## Supporting information

S1 Fig ZASC1 and ZBTB2 interact. (A) Schematic of ZBTB2 and relative location of N-terminal and internal deletion variants tested for interaction. Blue indicates interaction and red indicates a failure to interact. The location of zinc fingers are indicated in yellow. Sequences of the interaction domains in ZBTB2 zinc finger 1 are shown below the protein map, with Cys and His residues implicated in Zn^2+^ coordination indicated in green. The ZBTB2 mZF1 variant has C256S, C259S, H272A, H276A mutations. HEK293 cells (1X10^7^) were transfected with expression plasmids encoding the epitope tagged forms of the indicated proteins. 48 h post-transfection, cells were lysed and epitope tagged proteins were immunoprecipitated (IP), separated by SDS-PAGE and analyzed by western blotting (WB) using the indicated antibodies as described in materials and methods. (B) Co-immunoprecipitation of ZBTB2 variants by WT ZASC1.
(EPS)

S2 Fig Deletion of ZASC1 and ZBTB2 do not affect HIV integration. (A) 2LTR circle analysis was performed on indicated Jurkat cells (1X10^6^) challenged at an MOI of 1 with WT and ΔVpr replication competent HIV-1. Total cellular DNA was isolated and HIV 2LTR circle DNA and the cellular PDGB1 gene was quantitated by real-time Quantitative PCR. (B) Integration of VPR deletion virus is unaffected by ZASC1 and ZBTB2 deletion. Cells (1X10^6^) were challenged at an MOI of 5 (based on GFP levels in HEK293 cells) of VSV-G pseudotyped HIV-1 vector with nonsense mutations in envelope and Vpr, GFP-Nanoluc in the NEF locus and passaged for 7 days to allow for loss of unintegrated DNA. Total cellular DNA was isolated from 1X10^6^ cells and HIV DNA was quantitated by real-time Quantitative PCR with the HIV promoter primer set used in ChiP experiments. Error bars indicate the standard deviation of the data in all panels. ANOVA analysis was performed and for P-values < 0.05 a Tukey’s HSD was performed and relevant P-values reported.
(EPS)

S3 Fig ZBTB2 interacts with ZASC1 but not SP1 or Vpr. HEK293 cells (1X10^7^) were transfected with expression plasmids encoding the epitope tagged forms of the indicated proteins. 48 h post-transfection, cells were lysed and epitope tagged proteins were immunoprecipitated (IP) with anti-Flag beads, separated by SDS-PAGE and analyzed by western blotting (WB) using the indicated antibodies as described in Materials and Methods. (A) Co-immunoprecipitation of Flag-ZBTB2 or Flag-ZASC1 with either myc-tagged ZASC1 or SP1. (B) Co-immunoprecipitation of Flag-ZBTB2 or Flag-ZASC1 with myc-tagged Vpr. N.S. indicates a non-specific cross-reacting band.
(EPS)
